# Quercetin ameliorates aflatoxin B_1_-induced non-canonical ferroptosis in ovine oocytes through the OPA1/ACSL4 pathway

**DOI:** 10.1186/s40104-026-01454-3

**Published:** 2026-06-17

**Authors:** Lang Li, Bin Liu, Zhenzhen Wang, Ruiwen Zhao, Xiong Xiao, Yongju Zhao, Xiaoyan Qiu

**Affiliations:** 1 Chongqing Key Laboratory of Herbivore Science, College of Animal Science and Technology, Southwest University, Chongqing, 400715 China; 2 College of Veterinary Medicine, Southwest University, Chongqing, 400715 China

**Keywords:** Aflatoxin B_1_, Non-canonical ferroptosis, Quercetin, Sheep oocytes

## Abstract

**Background:**

Aflatoxin B_1_ (AFB_1_), a potent mycotoxin, commonly contaminates feeds like maize and soybean, jeopardizing animal reproduction. Although AFB_1_ exposure is known to cause oxidative stress, immune activation, and cell death in oocytes and early embryos of several species, its effects on sheep remain unclear. This study aimed to investigate AFB_1_-induced damage in ovine oocytes and its underlying mechanisms. Quercetin (QT), a cost-effective flavonoid with antioxidant and anti-inflammatory properties, is potential to improve this damage. The mechanisms may involve non-canonical ferroptosis, an iron-dependent, lipid peroxidation-driven cell death pathway independent of canonical regulators GPX4 and TFR1.

**Methods:**

4D Fast DIA-based micro-scale quantitative proteomics was conducted to identify the target proteins, and then immunofluorescence, qPCR, and parallel reaction monitoring (PRM) were conducted for the expression validation of the target proteins, and a series of analyses combined with the inhibitor experiments were conducted for the functional validation of the target proteins, including assessments of mitochondrial function (membrane potential ΔΨm, distribution, ATP levels), mitochondrial morphology observation by transmission electron microscopy (TEM), lipid peroxidation detection (LPO imaging, ROS and GSH detection), Fe^2+^ detection, endoplasmic reticulum staining, and early apoptosis signaling detection.

**Results:**

OPA1 and ACSL4 are screened as the target proteins by micro-scale quantitative proteomics analysis, and immunofluorescence, qPCR and PRM validate their expression. Molecular docking reveals that there is an interaction of OPA1 and ACSL4, and QT exhibits stronger binding affinity to both OPA1 and ACSL4 than AFB_1_. AFB_1_ induces aberrant upregulation of OPA1 and ACSL4, disrupting mitochondrial cristae and membrane structure, impairing mitochondrial function and energy metabolism (including decreased mitochondrial membrane potential, abnormal distribution, and reduced ATP synthesis), promoting lipid peroxidation and Fe^2+^ accumulation, exacerbating endoplasmic reticulum stress and altering ROS/GSH levels, ultimately leading to ferroptosis in oocytes. Addition of QT ameliorates the AFB_1_-induced abnormal expression of OPA1 and ACSL4.

**Conclusions:**

QT alleviates AFB_1_-induced damage on ovine oocytes by suppressing non-canonical ferroptosis via the OPA1/ACSL4 pathway. These findings elucidate a novel mechanism underlying AFB_1_-mediated reproductive toxicity and provide a theoretical foundation for applying QT to mitigate AFB_1_-induced reproductive impairment and improve livestock health.

**Graphical Abstract:**

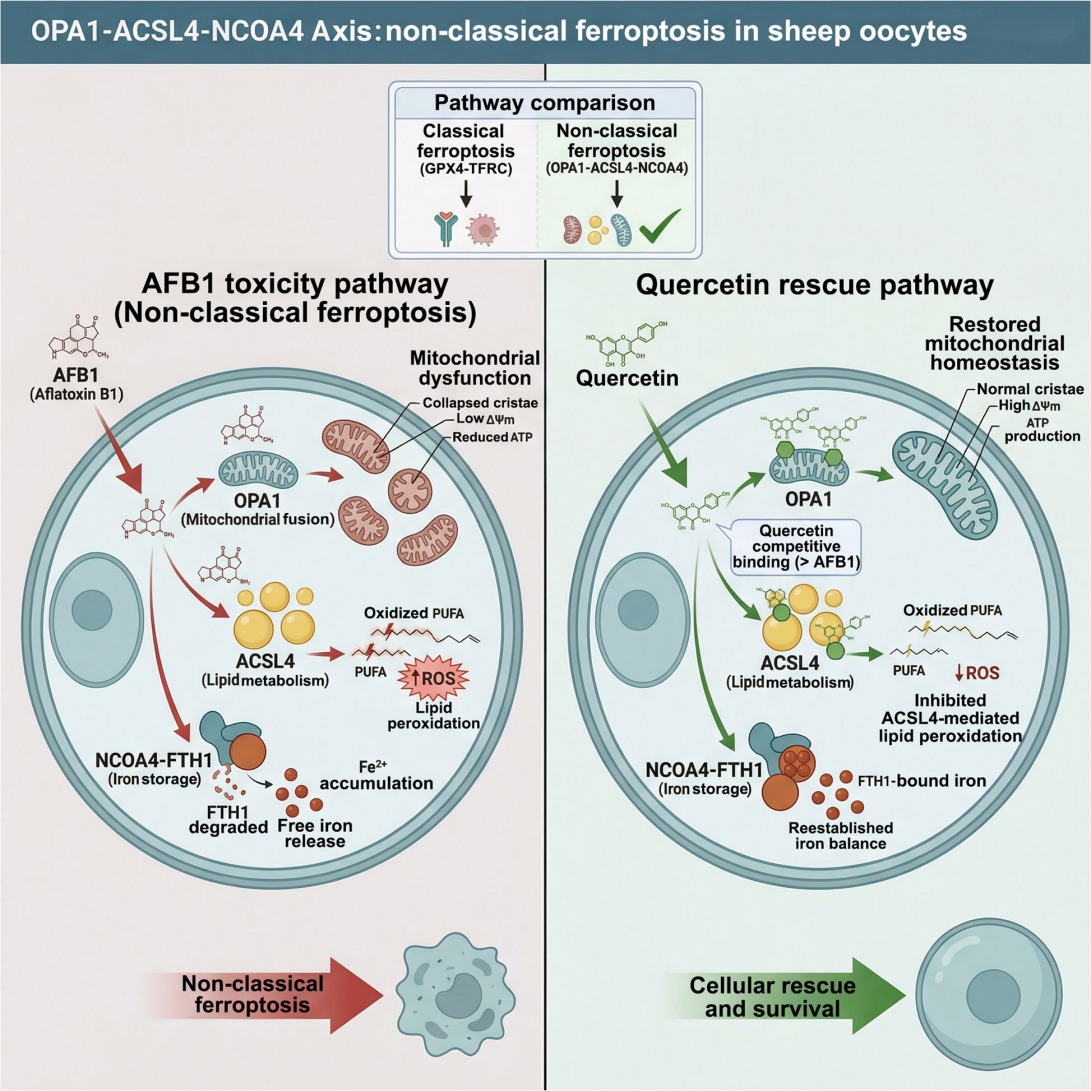

**Supplementary Information:**

The online version contains supplementary material available at 10.1186/s40104-026-01454-3.

## Introduction

Mycotoxins that have been widely studied include five major categories: aflatoxins (AFs), ochratoxin A (OTA), fumonisins (FB), zearalenone (ZEN), and deoxynivalenol (DON). Consuming grains such as corn, soybeans and millet, or their products that contain mycotoxins can have a serious impact on the health of both humans and animals [1, 2]. Among aflatoxins, aflatoxin B_1_ (AFB_1_) is the most toxic. Classified as a Group 1 carcinogen by the International Agency for Research on Cancer (IARC), AFB_1_ can persist in the body in molecular form and is a major cause of hepatocellular carcinoma [3]. It is estimated that approximately 25% of agricultural products are contaminated with AFB_1_ annually, with some reports suggesting contamination rates may reach 60%–80% [4]. Dietary exposure to AFB_1_ through contaminated feed grains significantly impairs animal growth performance and reproductive capacity [5].

Research in mice has demonstrated that AFB_1_ can be directly absorbed into the bloodstream as AFB_1_ molecules in the stomach and intestines [6]. Similarly, studies in sheep have shown that AFB_1_ is absorbed through the gastrointestinal system into the thoracic duct lymph or venous plasma, where it directly acts on body tissues and cells, causing widespread systemic harm [7]. Feeding zebrafish with AFB_1_-contaminated feed compromises the animals' reproductive performance, including oocyte quality [8]. AFB_1_ enters cells and is metabolically activated by cytochrome P450 to form aflatoxin B_1_−8,9-epoxide (AFBO). AFBO binds to DNA and proteins, forming adducts that cause DNA strand breaks and gene mutations, and damage protein structure and function, thereby impairing cell activity and bodily functions [8, 9]. Moreover, AFBO induces oxidative stress, leading to lipid peroxidation that disrupts cell structure and function [8, 9]. Existing evidence indicates that AFB_1_ exposure adversely affects germ cells across multiple species. Studies in mice, pigs, and cattle show that AFB_1_ induces damage in oocytes, granulosa cells, early embryonic cells, and ovarian tissue, primarily through oxidative stress, immune reactions, and apoptotic or necrotic cell death [10–12]. For instance, in healthy female rats, sustained AFB_1_ exposure over 21 days caused significant follicular atresia, cytoplasmic and nuclear degeneration in oocytes, thinning of the zona pellucida, and granulosa cell deterioration, ultimately reducing or depleting ovulatory follicles [13]. During in vitro maturation of pig oocytes, AFB_1_ exposure compromised nuclear and cytoplasmic maturation in a dose- and time-dependent manner, leading to poor blastocyst quality. It also promoted reactive oxygen species (ROS) accumulation, induced apoptosis, disrupted the glutathione/GPX system, and inhibited mitochondrial DNA replication and biogenesis [10]. A preliminary study by Hajarizadeh et al. [14] on sheep oocytes revealed that AFB_1_ reduced the meiosis II (MII) maturation rate, decreased intracellular glutathione (GSH) levels, and significantly increased ROS levels. However, that study remained descriptive, lacking investigation into mitigation strategies or deeper mechanistic insights.

Ferroptosis is a regulation form of cell death driven by iron overload and dysregulated metabolism of polyunsaturated fatty acids [15]. Fe^2+^ initiate lipid peroxidation via the Fenton reaction. The resulting lipid peroxides can further damage iron-regulatory proteins or promote iron release, thereby increasing free Fe^2+^ levels and creating a self-sustaining vicious cycle [16]. This process is often accompanied by cellular oxidative stress, marked by elevated reactive oxygen species (ROS) and reduced glutathione (GSH) levels [17]. Excess ROS attack polyunsaturated fatty acids (PUFAs), inducing lipid peroxidation [18]. Lipid peroxidation is an oxidative deterioration of lipids or PUFAs resulting from a free radical chain reaction under oxidative stress [19]. Consequently, oxidative stress promotes ROS generation and lipid peroxidation. Catalysed by iron ions, this process oxidises PUFAs in the cell membrane to lipid hydroperoxides, thereby triggering and accelerating ferroptosis [18, 19]. Mitochondrial dysfunction directly drives ferroptosis through multiple mechanisms. Mitochondrial impairment markedly elevates intracellular ROS, exacerbating lipid peroxidation. It also disrupts cellular energy metabolism, increasing sensitivity to oxidative stress. Moreover, mitochondrial malfunction induces accumulation of the labile iron pool. The surplus iron ions catalyse lipid peroxidation and activate GPX4-independent ferroptosis pathways, ultimately leading to cell membrane damage and cell death [18–20]. Evidence indicates that AFB_1_ disrupts iron homeostasis and induces ferroptosis in mammalian cells [21, 22]. AFB_1_ exposure significantly lowers the levels of the antioxidant enzymes superoxide dismutase (SOD), catalase (CAT), glutathione peroxidase (GSH-Px), and glutathione (GSH), while markedly increasing the accumulation of transferrin receptor 1 (TFR1), ferritin heavy chain 1 (FTH1), solute carrier family 3 member 2 (SLC3A2), and Fe^2+^ concentration. This leads to oxidative stress and lipid peroxidation, ultimately inducing cellular ferroptosis [8, 11]. Wang et al. [8] reported that AFB_1_ exposure in zebrafish alters the expression of apoptosis, inflammation, and ferroptosis related molecules in liver, ovary, and embryonic tissues, promoting ferroptosis through suppression of GPX4 and SLC7A11 expression. Although ferroptosis is gaining attention in reproductive biology, its role in AFB_1_-induced reproductive damage in sheep remains unclear.

AFB_1_ is highly thermostable, withstanding temperatures up to 260 °C, which hinders its removal from animal feed grains and related products, posing a serious threat to animal health [23]. To date, no highly effective detoxifying agent against AFB_1_ is available. Studies in pigs have shown that antioxidants such as isorhamnetin, melatonin, and curcumin can alleviate AFB_1_-induced toxicity [2, 10, 11]. Compared to these three agents, QT exhibits broader therapeutic potential, including antioxidant, anti-inflammatory, antimicrobial, immunomodulatory, and neuroprotective properties, and more importantly, being more cost-effective [24]. In the field of cell death regulation, the anti-ferroptotic effect of quercetin has become a growing research focus. Studies indicate that quercetin inhibits ferroptosis through multiple pathways: it upregulates antioxidant enzymes like GPX4, modulates iron metabolism-related proteins (e.g., hepcidin and ferritin) to reduce intracellular free iron accumulation, thereby blocking harmful ROS generation via the Fenton reaction and enhancing cellular clearance of lipid peroxides [9, 25, 26]. In humans and mice, QT has been reported to reduce oxidative stress and apoptosis in oocytes by restoring mitochondrial function and decreasing ROS, thereby improving oocyte quality and in vitro maturation efficiency [27, 28]. However, its protective role in sheep oocytes exposed to AFB_1_ remains unexplored. This study aims to investigate the beneficial effects of QT on AFB_1_-induced damage in sheep oocytes and to elucidate the underlying mechanisms.

Optic atrophy 1 (OPA1), a dynamin-like GTPase located in the mitochondrial inner membrane, regulates key processes including mitochondrial fusion, electron transport chain supercomplex assembly, cristae remodeling, mitochondrial DNA maintenance, and energy metabolism [29]. Meanwhile, acyl-CoA synthetase long-chain family member 4 (ACSL4) enhances cellular susceptibility to ferroptosis by catalyzing the esterification of long-chain polyunsaturated fatty acids (PUFAs), such as arachidonic acid (AA) and adrenic acid (AdA), into PUFA-CoAs. These esters are subsequently incorporated into membrane phospholipids, forming substrates that are highly prone to lipid peroxidation and thus promoting ferroptosis [30, 31]. Recent studies have begun to link OPA1 to the regulation of ferroptosis. For example, Liang et al. [32] demonstrated that OPA1 deficiency confers strong resistance to ferroptosis in both human and mouse cells. Similarly, Shi et al. [33] reported that chlorantraniliprole (CAP) induces mitophagy, ferroptosis, and cytokine homeostasis imbalance in grass carp hepatocytes by disrupting the expression of both OPA1 and ACSL4. OPA1 drives ACSL4-mediated lipid peroxidation and Fe^2+^ accumulation to induce ferroptosis by promoting mitochondrial lipid ROS generation and inhibiting the ATF4-mediated integrated stress response [30–32]. This ferroptosis depends on its GTPase activity and is independent of OPA1-mediated mitochondrial fusion [32]. Although these findings suggest that OPA1 and ACSL4 are both involved in ferroptosis, it remains unclear whether they interact to co-regulate this process and also unclear whether they have effects on sheep oocytes.

Therefore, we propose that AFB_1_ exposure disrupts oocyte mitochondrial function and structure via the OPA1/ACSL4 pathway, inducing oxidative stress, lipid peroxidation, inflammatory response, and ferroptosis, whereas QT supplementation alleviates these effects. Our results demonstrate that AFB_1_ induces non-canonical ferroptosis, as confirmed by proteomics, PRM validation, and the effect of the ferroptosis inhibitor Fer-1. The OPA1/ACSL4 pathway participates in QT-mediated regulation of mitochondrial function and ferroptosis. These findings extend the mechanistic understanding of AFB_1_ and QT action on animal germ cells and provide a theoretical basis for using QT to mitigate AFB_1_-induced reproductive damage and improve animal health.

## Materials and methods

### Oocyte retrieval and culture of cumulus oocyte complexes (COCs)

Ovaries were collected from a local abattoir and transported to the laboratory in phosphate-buffered saline (PBS) containing antibiotics at 25–35 °C. Upon arrival, the ovaries were rinsed three times with antibiotic-supplemented PBS, gently dried with sterile gauze, and stabilized for further processing. Cumulus-oocyte complexes (COCs) were released from 2–6 mm follicles by either puncturing or aspirating using a sterile surgical blade or a 10-mL syringe needle. Only COCs exhibiting homogeneous cytoplasm, dark coloration, and at least three compact layers of cumulus cells were selected under a stereomicroscope (SD-101, Olympus, Tokyo, Japan). The chosen COCs were washed three times in pre-warmed M199 complete medium. For in vitro maturation (IVM), 10–30 COCs were evenly distributed into six-well plates, each well containing 500 μL of M199 complete medium overlaid with mineral oil, and cultured for 23 h at 38.5 °C under 5% CO_2_ in a humidified atmosphere. The M199 complete medium consisted of 10% fetal bovine serum (FBS), 1 μg/mL 17β-estradiol, 10 IU/mL pregnant mare serum gonadotropin (PMSG/eCG), 10 IU/mL human chorionic gonadotropin (hCG), 10 ng/mL epidermal growth factor (EGF), 100 IU/mL penicillin, and 100 μg/mL streptomycin.

AFB_1_ was dissolved in dimethyl sulfoxide (DMSO) to prepare a 1 mg/mL stock solution, which was then diluted in M199 complete medium to final concentrations of 5, 15, and 30 μmol/L for oocyte treatment. Similarly, QT was prepared as a 1 mg/mL stock in DMSO and diluted in M199 medium to concentrations of 10, 20, and 40 μmol/L. The final DMSO concentration in the maturation medium was kept below 1% and was consistent across all experimental groups.

After 23 h of maturation, cumulus cells were removed from oocytes using 0.1% hyaluronidase. The oocytes were then stained with Hoechst 33342 for 30 min, washed three times with PBS, and examined under an inverted fluorescence microscope (TE2000-U, Nikon, Tokyo, Japan). Oocyte maturation was identified by the extrusion of the first polar body (Pb1). The oocytes were allocated to three treatment groups: control, AFB_1_, and AFB_1_ + QT. Each treatment group included three replicates, with 25–35 oocytes per replicate.

### 4D Fast DIA micro scale quantitative proteomic analysis

Microscale quantitative proteomic analysis was performed using 4D Fast DIA at Jingjie PTM BioLab Co., Ltd. (Hangzhou, China). Proteins were extracted, digested with trypsin, and analyzed by LC-MS/MS. Bioinformatic processing followed data acquisition. Raw data were processed using DIA-NN (v1.8) for database searching against the Uniprot reference database supplemented with a common contaminant database, with a false discovery rate (FDR) threshold of ≤ 1% for protein identification. Proteins were required to be supported by ≥ 2 unique peptides; shared peptides and low-abundance signals were excluded. Data were log_2_-transformed and normalised by median centring. Inter-batch variation was adjusted using ComBat, and retention time was aligned using iRT peptides. Technical replicates (*n* = 3) were included in the LC‑MS/MS workflow. System stability was monitored by injecting pooled quality control (QC) samples in each batch. Only proteins with a coefficient of variation (CV) ≤ 20% across technical replicates were retained for downstream analysis. Peptide separation was carried out on a NanoElute ultra-high-performance liquid chromatography system. Ionization was achieved via a capillary ion source, and analysis was performed on a timsTOF Pro mass spectrometer (Bruker, USA) equipped with trapped ion mobility spectrometry (tims) and time-of-flight (TOF) detection. Each treatment group included three replicates, with 15 oocytes per replicate.

### LC-MS/MS analysis

Tryptic peptides were dissolved in 0.1% formic acid (solvent A) and loaded onto a self-packed reversed-phase analytical column. Using an EASY-nLC 1000 UPLC system, a gradient was run at a constant flow rate of 700 nL/min as follows: solvent B (98% acetonitrile, 0.1% formic acid) was increased from 6% to 23% over 38 min, raised to 35% in 14 min, then elevated to 80% within 4 min and held for 4 min.

 Peptides were ionized via an NSI source and analyzed by tandem mass spectrometry (MS/MS) on a Q Exactive™ Plus instrument (Thermo) coupled online to the UPLC system. The electrospray voltage was set to 2.0 kV. Full-scan MS spectra (*m/z* 350–1,000) were acquired in the Orbitrap at a resolution of 35,000. MS/MS scans were performed with a normalized collision energy of 27, and fragments were detected in the Orbitrap at a resolution of 17,500. A data-independent acquisition (DIA) method was used, comprising one full MS scan followed by 20 MS/MS scans. Automatic gain control (AGC) targets were set to 3E6 for MS and 1E5 for MS/MS. Maximum injection times were 20 ms for full MS and set to auto for MS/MS. The isolation window for MS/MS was 2.0 *m/z*.

### Targeted proteomics quantitative detection (PRM)

Parallel reaction monitoring (PRM) mass spectrometry provides high interference resistance and maintains elevated detection sensitivity even in complex matrices. This technique was employed to quantitatively validate candidate proteins identified via 4D Fast DIA-based microscale quantitative proteomic screening. For protein extraction, the sample was ground in liquid nitrogen to generate a fine cell powder, which was transferred to a 5-mL centrifuge tube. Lysis buffer (8 mol/L urea, 1% Triton-100, 10 mmol/L dithiothreitol, and 1% protease inhibitor cocktail) was added at a 4:1 volume-to-powder ratio. The mixture was sonicated three times on ice using a high-intensity ultrasonic processor (Scientz). After centrifugation at 20,000 × *g* and 4 °C for 10 min, the supernatant was collected. Proteins were precipitated with cold 20% trichloroacetic acid at −20 °C for 2 h, followed by centrifugation at 12,000 × *g* and 4 °C for 10 min. The pellet was washed three times with cold acetone and redissolved in 8 mol/L urea. Protein concentration was determined using a BCA kit according to the manufacturer’s instructions. PRM validation was performed based on protein expression data acquired through 4D Fast DIA-based micro-scale quantitative proteomics, with three biological replicates per treatment group and 15 oocytes analysed per replicate.

### Molecular docking

For small molecule-protein docking, the 3D structures of quercetin (PubChem CID: 5280343) and aflatoxin B_1_ (PubChem CID: 186907) were obtained from PubChem. Ligand structures were energy-minimized with Chem3D and converted to PDBQT format using AutoDock Tools. The protein structures of OPA1 (AlphaFold ID: AF-O60313-F1-v6) and ACSL4 (AlphaFold ID: AF-O60488-F1-v6) were retrieved from UniProtKB. Using PyMOL 3.0.3, water molecules and non-essential ligands were removed, and hydrogen atoms were added. Molecular docking was performed with AutoDock Vina 1.2.7. The grid box for OPA1 was centred at (4.717, 3.462, −6.658) with dimensions 171.04 × 98.7 × 134.18 Å; for ACSL4, the centre was (0.354, 0.129, −3.531) with dimensions 79.83 × 84.434 × 86.28 Å. An exhaustiveness of 10 was used for both proteins, with other parameters set to default. Nine binding poses were generated for each ligand–protein pair. The conformation with the lowest binding energy and highest cluster frequency was selected as the optimal binding mode. Complex structures were visualized in PyMOL 3.0.3, and interaction diagrams were created with Discovery Studio.

For protein–protein docking, rigid-body docking was conducted using HDOCK. The 3D structures of OPA1 and ACSL4 were prepared by removing water molecules, adding hydrogens, and performing a brief energy minimization in PyMOL, then saved in PDB format. Docking was performed with default parameters, and the top 10 poses (ranked by binding affinity) were obtained. The optimal complex was selected based on interaction surface area, hydrogen bonds, and key residue contacts, and visualized using PyMOL 3.1. Specific docking settings included a grid spacing of 1.200, angle step of 15.000, and initial rotation of (0.00000, 0.00000, 0.00000). The geometric centre for OPA1 in the docking grid was (1.338, 0.478, −1.861), and for ACSL4 it was (1.125, 5.417, −5.918). The resulting REMARK Score was −292.97 and REMARK RMSD was 69.99.

### Immunofluorescence staining

Oocytes were fixed in 4% paraformaldehyde for 30 min, permeabilized with immunostaining buffer for 10 min, and blocked with 2% bovine serum albumin (BSA) for 1 h. Subsequently, samples were incubated overnight at 25 °C with primary antibodies against OPA1 (1:50) (A9833, ABclonal Technology, China), ACSL4 (1:50) (A6826, ABclonal Technology, China), and FTH1 (1:50) (A25458PM, ABclonal Technology, China). After washing, oocytes were incubated with corresponding secondary antibodies (AF555‑conjugated donkey anti‑rabbit IgG (H + L), A0453, Beyotime Biotechnology, Shanghai, China) at 37 °C for 1 h. Fluorescence signals were assessed using a fluorescence microscope, and images were analyzed with ImageJ software (National Institutes of Health, Bethesda, MD, USA). The mean gray value for each oocyte was calculated by dividing the integrated optical density by its corresponding area. Each treatment group included three replicates, with 10–15 oocytes per replicate.

### Mitochondrial membrane potential (MMP) detection

The mitochondrial membrane potential (MMP, ΔΨm) of oocytes was assessed using the JC-1 assay kit (C2006, Beyotime Biotechnology, Shanghai, China). After incubation in a pre-equilibrated JC-1 working solution at 37 °C for 30 min, fluorescence images were captured under an inverted fluorescence microscope. The red/green fluorescence intensity ratio was quantified for each oocyte using ImageJ software, reflecting the relative ΔΨm value. Each treatment group included three replicates, with 15–25 oocytes per replicate.

### Mitochondrial distribution assessment

To evaluate mitochondrial distribution, oocytes were stained with MitoTracker^®^ Red CMXRos (M9940, Solarbio, Beijing, China) for 30 min at 37 °C. After washing, the samples were examined under a fluorescence microscope. Mitochondria showing a homogeneous staining pattern were considered normal, whereas those exhibiting aggregated, intermediate, or faint signals were classified as abnormal. Each treatment group included three replicates, with 10–15 oocytes per replicate.

### ATP content measurement

The ATP content in oocytes was measured with the mitochondrial fluorescent probe pCMV-Mito-AT1.03 (D2606, Beyotime Biotechnology, Shanghai, China). Following a 12-h incubation at 38.5 °C in a pre-equilibrated working solution (0.1 µg/µL), MII-stage oocytes were examined using an inverted fluorescence microscope. Each treatment group included three replicates, with 10–15 oocytes per replicate.

### LPO imaging

Lipid peroxidation (LPO) in oocytes was detected using the fluorescent probe BODIPY 581/591 C11 (S0043S, Beyotime Biotechnology, Shanghai, China). Oocytes were incubated with 2 μmol/L of the probe at 37 °C for 30 min, and fluorescence signals were visualized under an inverted fluorescence microscope. Each treatment group included three replicates, with 15–20 oocytes per replicate.

### Fe^2+^ detection

The intracellular ferrous iron (Fe^2+^) level in oocytes was measured using a Cell Ferrous Iron Fluorometric Assay Kit (CA1530, Solarbio, Beijing, China). Oocytes were incubated with a 6 μmol/L fluorescent probe at 37 °C for 40 min, and Fe^2+^-specific fluorescence was observed under an inverted fluorescence microscope. Each treatment group included three replicates, with 10–15 oocytes per replicate.

### ROS and GSH detection

Intracellular ROS levels were measured using the fluorescent probe 2′,7′-dichlorodihydrofluorescein diacetate (DCFH-DA; E004-1-1, Nanjing Jiancheng, China). Oocytes were incubated with 10 μmol/L DCFH-DA in serum-free medium at 37 °C for 20 min, and fluorescence was observed under an inverted fluorescence microscope. Each treatment group included three replicates, with 10–20 oocytes per replicate.

The glutathione (GSH) content was assessed with CellTracker Blue CMF2HC (HY-D1571, MCE, USA). Oocytes were stained with 10 μmol/L of the probe at 38 °C for 30 min, and fluorescence signals were examined under an inverted fluorescence microscope. Each treatment group included three replicates, with 10–20 oocytes per replicate.

### Endoplasmic reticulum staining

Oocytes were stained with ER-Tracker Blue (R32854, Yuanye, Shanghai, China) for 30 min at 37 °C to visualize the endoplasmic reticulum. After washing, ER distribution was assessed by fluorescence microscopy using the same classification criteria as for mitochondria (Sect. "Mitochondrial distribution assessment"). Each treatment group included three replicates, with 15–20 oocytes per replicate.

### Early apoptosis detection

To assess apoptosis, oocytes were first incubated for 20 min at 38.5 °C in 100 μL binding buffer supplemented with 2.5 μL Annexin V-FITC (Beyotime Biotechnology, Shanghai, China; Cat. No. C1062). Following this, the oocytes were washed three times with PBS and observed under an inverted fluorescence microscope. Each treatment group included three replicates, with 10–20 oocytes per replicate.

### Transmission electron microscopy (TEM)

For transmission electron microscopy, samples were initially fixed in 3% glutaraldehyde and subsequently post-fixed with 1% osmium tetroxide. Following gradient dehydration and resin infiltration, the tissues were trimmed, re-embedded, and sectioned. Finally, the sections were stained with uranyl acetate and lead citrate and observed under a JEM-1400FLASH TEM (JEOL, Japan). Each treatment group included three replicates, with 15–20 oocytes per replicate.

### Data analysis

#### 4D Fast DIA data analysis

The data from DIA were analyzed using the DIA-NN search engine (version 1.8). Tandem mass spectra were matched against the Capra_hircus_9925_PR_20240925.fastadatabase (32,608 entries) supplemented with a reverse decoy database. Trypsin/P was specified as the protease, allowing up to one missed cleavage. Fixed modifications included N-terminal methionine excision and cysteine carbamidomethylation. The false discovery rate (FDR) was controlled at < 1%. Reproducibility within sample groups was assessed using Pearson correlation, principal component analysis (PCA), and relative standard deviation. Differential proteins were identified after quality control, and relative quantitative changes between groups were evaluated by a *t*-test. A significance threshold of *P* ≤ 0.05 was applied, with protein fold-change > 1.2 considered up-regulated and < 0.8 down-regulated.

Subcellular localization was predicted using WoLF-PSORT. Gene Ontology (GO) terms were assigned via eggnog-mapper and the EggNOG database, with functional categorization covering cellular component, molecular function, and biological process. Protein domain annotation was performed using the Pfam database and PfamScan. Pathway analysis was conducted by aligning proteins to the KEGG database via BLAST (blastp, E-value ≤ 1 × 10^−^^4^). Enriched pathways among differentially expressed proteins (DEPs) were identified by a two-tailed Fisher’s exact test, with enrichment fold > 1.2 and *P* ≤ 0.05 considered significant. Functional terms significantly enriched among DEPs (*P* < 0.05) were clustered by one-dimensional hierarchical clustering after −log transformation of *P*-values, and results were visualized as a heatmap using the ComplexHeatmap R package. Protein–protein interaction (PPI) networks were constructed by submitting DEP accessions or sequences to the STRING database, retaining only interactions present in the retrieved dataset. Interactions with a confidence score > 0.7 were considered high-confidence and visualized using the visNetwork R package. All experiments were repeated independently at least three times. Each treatment group included three replicates, with 15 oocytes per replicate.

#### *PRM* analysis

Mass spectrometry data were processed with Skyline (v3.6). Trypsin [KR/P] was specified as the enzyme, allowing up to 2 missed cleavages. Peptide length was set to 8–25 amino acids. Variable modifications included carbamidomethylation (Cys) and oxidation (Met), with a maximum of 3 modifications per peptide. Precursor charges were set to 2 and 3; ion charges to 1 and 2; ion types included b, y, and p. Product ions were considered from ion 3 to the final ion, with an ion match tolerance of 0.02 Da. Each treatment group included three replicates, with 15 oocytes per replicate.

Statistical analysis was performed using GraphPad Prism (version10.0) and SPSS (version26.0). Data are expressed as mean ± SEM. Differences between groups were assessed by one-way ANOVA followed by Duncan’s post hoc test. A *P*-value < 0.05 was considered statistically significant. All experiments included three biological replicates.

## Results

### Selection of AFB_1_ and QT concentrations

**Fig. 1:**
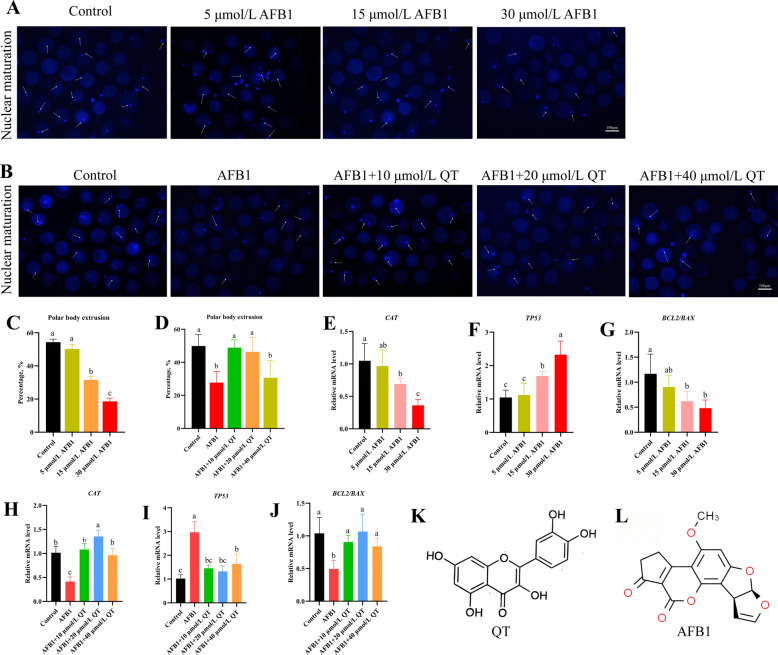
Effects of AFB_1_ on nuclear maturation of sheep oocytes and the ameliorative effect of QT. Oocytes were subjected to co-culture with varying concentrations of AFB_1_ (5, 15, and 30 μmol/L) alongside or without different levels of QT (10, 20, and 40 μmol/L) for a duration of 23 h. **A** Outcomes of nuclear maturation in oocytes exposed to various concentrations of AFB_1_ are presented. The mature oocyte is highlighted by a white arrow. Scale bar: 100 μm. **B** The protective influence of distinct QT concentrations on the reduction of oocyte nuclear maturation caused by 15 μmol/L AFB_1_ is depicted. Scale bar: 100 μm. **C** A statistical evaluation of the Pb1 extrusion rates in oocytes treated with different AFB_1_ concentrations. *n* = 75. **D** Statistical findings regarding Pb1 extrusion rates for oocytes subjected to varying doses of QT in conjunction with 15 μmol/L AFB_1_. *n* = 75. **E**–**G** *CAT, TP53 *and* BCL2/BAX* mRNA expression levels in the control group and those exposed to 5, 15, and 30 μmol/L AFB_1_. **H**–**J** *CAT, TP53 *and* BCL2/BAX* mRNA levels measured in the control, AFB_1_, and AFB_1_ with 10, 20, and 40 μmol/L QT. **K** Molecular configuration of quercetin. **L** Molecular configuration of aflatoxin B_1_. The letter “*n*” indicated the total number of oocytes in each group of three independent replicates. ^a−c^Values marked with different superscripts denote statistical significance (*P* < 0.05). The data are represented as mean ± SEM

Oocyte maturation rates did not differ significantly between the control group and the group exposed to 5 μmol/L AFB_1_ (*P* > 0.05; Fig. 1A and C). In contrast, exposure to 15 and 30 μmol/L AFB_1_ significantly reduced maturation rates in a dose-dependent manner (*P* < 0.05; Fig. 1A and C), with rates declining to 31.58% ± 2.09% and 18.56% ± 2.01%, respectively. Furthermore, oocytes treated with 15 or 30 μmol/L AFB_1_ showed significantly lower expression of *CAT* and the *BCL2/BAX* ratio, along with a marked increase in *TP53* expression compared to the control (*P* < 0.05; Fig. 1E–G). Based on these findings, 15 μmol/L AFB_1_ was selected for subsequent experiments.

As shown in Fig. 1B and D, AFB_1_ exposure significantly decreased the oocyte nuclear maturation rate compared with the control group (*P* < 0.05). Although no significant improvement was observed with 40 μmol/L QT (*P* > 0.05), both 10 μmol/L and 20 μmol/L QT significantly increased the nuclear maturation rate to 48.89% ± 1.11% and 46.34% ± 2.06%, respectively (*P* < 0.05). Furthermore, 10 μmol/L QT markedly upregulated *CAT* and *BCL2/BAX* expression while suppressing *TP53* levels relative to the AFB_1_-treated group (*P* < 0.05; Fig. 1H–J). Based on these results, 10 μmol/L QT was selected for the following experiments.

### Identification and quantitative analysis of proteins

**Fig. 2:**
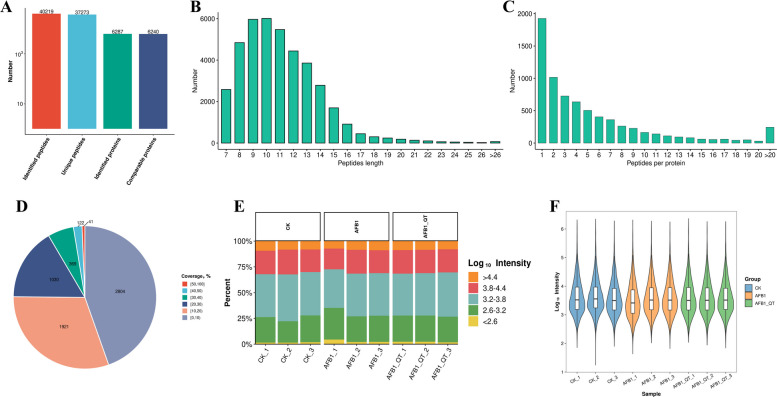
Identification and quantitative analysis of proteins. **A** Overview of protein identification. **B** Peptide length distribution. **C** Peptide number distribution. **D** Protein coverage distribution. **E** Histogram of protein intensity distribution. **F** Violin plot of protein intensity values

4D Fast DIA-based micro-scale quantitative proteomics was performed to compare protein profiles among the control, AFB_1_, and AFB_1_ + QT groups. A total of 40,219 peptides corresponding to 37,273 unique peptides were identified, leading to the quantification of 6,240 out of 6,287 detected proteins (Fig. 2A). The identified peptides ranged from 7 to 20 amino acids in length (Fig. 2B), consistent with typical tryptic digestion and MS fragmentation patterns. Most proteins were supported by at least two peptides (Fig. 2C), improving quantitative accuracy. Protein sequence coverage was generally below 30% (Fig. 2D), which is expected in shotgun proteomics where detection correlates with abundance. Over 92.5% of proteins had an abundance value exceeding 2.6 (Fig. 2E). Violin plots of protein intensities showed comparable distributions across all samples (Fig. 2F), indicating high data quality and reproducibility. Together, these results confirm the reliability of the proteomic data generated in this study.

### Differential protein screening and bioinformatics analysis

**Fig. 3:**
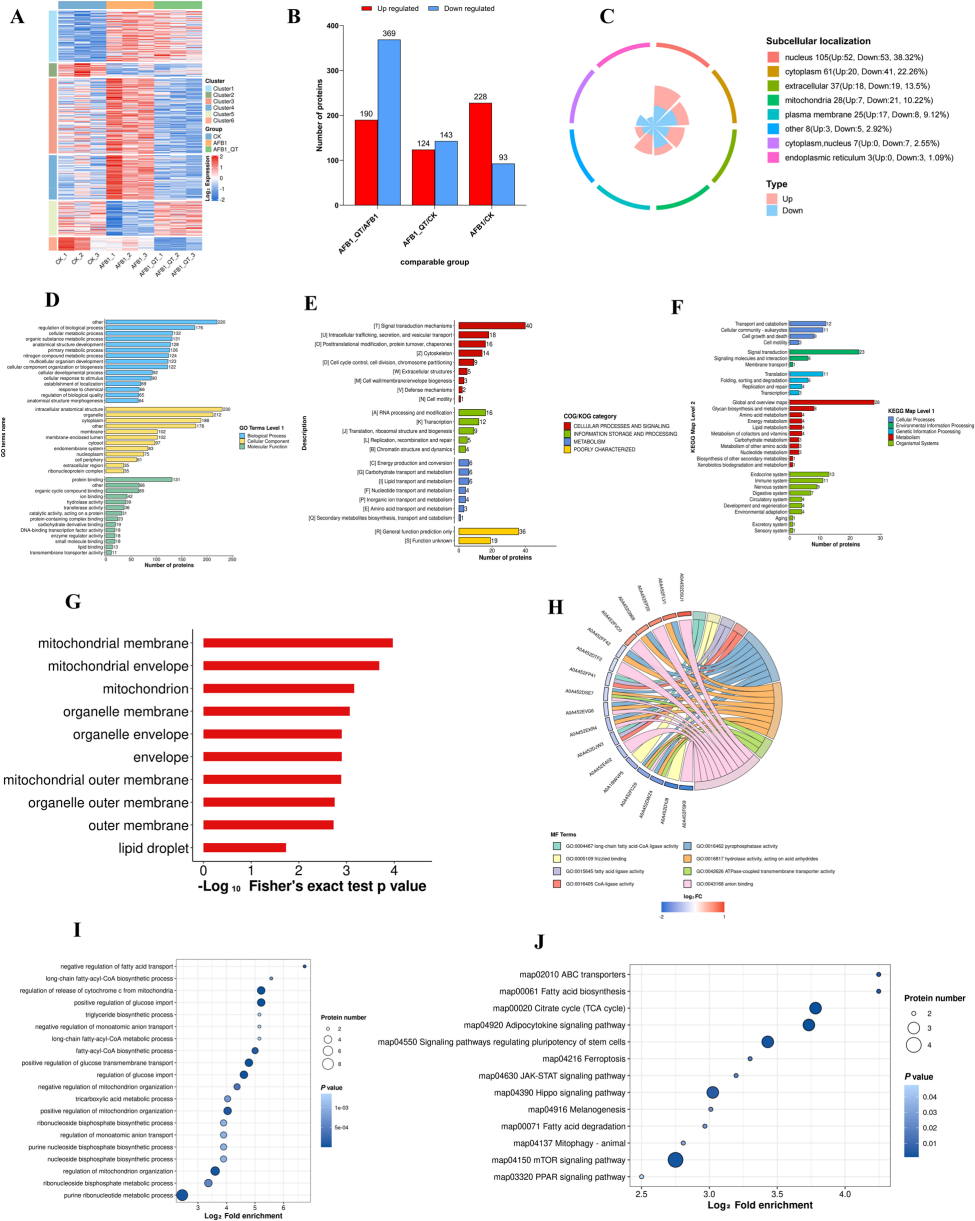
Differential protein screening and bioinformatics analysis. **A** Analysis of clustering for proteins with differential expression across groups. **B** Statistical representation of proteins differentially expressed. **C** Classification of subcellular localization for differentially expressed proteins. **D** Secondary GO classification of proteins with differential expression. **E** Functional classification of differentially expressed proteins categorized by COG/KOG. **F** KEGG-based functional classification of proteins. **G** Enrichment of differentially expressed proteins within protein domains. **H** Enrichment of differentially expressed proteins in terms of molecular functions. **I** Enrichment of differentially expressed proteins relating to biological processes. **J** Enrichment of differentially expressed proteins in relation to KEGG pathways

Differentially expressed proteins (DEPs) were identified using a fold change (FC) threshold of > 1.20 or < 0.80 and a *P*-value of < 0.05 (Fig. 3A–J). To visualize expression patterns, k-means clustering was applied to proteins with an ANOVA *P*-value < 0.05, and a heatmap was generated accordingly. The heatmap displays sample groups on the horizontal axis and DEPs on the vertical axis, with a color gradient from red (high expression) to blue (low expression). Three biological replicates showed consistent coloring, indicating high reproducibility (Fig. 3A). Most DEPs exhibited similar expression levels between the control and AFB_1_ + QT groups but differed markedly from the AFB_1_ group (Fig. 3A). In total, 559 DEPs (190 upregulated, 369 downregulated) were identified between the AFB_1_ + QT and AFB_1_ groups, 321 DEPs (228 upregulated, 93 downregulated) between the AFB_1_ and control groups, and 267 DEPs (124 upregulated, 143 downregulated) between the AFB_1_ + QT and control groups (Fig. 3B).

Subcellular localization analysis indicated that the DEPs between the AFB_1_ + QT and AFB_1_ groups were primarily localized to the nucleus, cytoplasm, mitochondria, and cell membrane, collectively accounting for 79.92% of all DEPs (Fig. 3C). Gene Ontology (GO) classification grouped these DEPs into three major categories: Biological Process, Cellular Component, and Molecular Function (Fig. 3D). Within Cellular Component, DEPs were mainly associated with organelles, cytoplasm, and membranes. Their molecular functions included protein binding, ion binding, hydrolase and transferase activities, carbohydrate derivative binding, DNA-binding transcription factor activity, lipid binding, and transmembrane transporter activity. These DEPs were involved in biological processes such as cellular metabolism, organic and nitrogen compound metabolism, cellular development, translation, and cellular responses to stimuli and chemicals (Fig. 3D).

COG/KOG classification indicated that the DEPs were primarily associated with metabolism, information storage and processing, and cellular processes/signaling (Fig. 3E). KEGG pathway enrichment analysis further revealed that these DEPs were mainly involved in cellular functions (including transport, catabolism, proliferation, apoptosis, and motility), signal transduction, and multiple metabolic processes (such as amino acid, lipid, carbohydrate, and energy metabolism). These DEPs were also enriched in pathways related to organismal systems, such as the endocrine, immune, and nervous systems, as well as environmental adaptation (Fig. 3F).

Based on the integrated proteomic results, DEPs were identified in processes including mitochondrial function, lipid metabolism, ATP energy metabolism, general metabolism, cellular stress, endoplasmic reticulum, and membrane structures. Functional enrichment analysis was subsequently performed on DEPs related to mitochondrial function, lipid metabolism, ATP energy metabolism, cellular stress, endoplasmic reticulum, and membrane structures, filtered using thresholds of fold change (FC) > 1.20 or < 0.80 and *P* < 0.05. Subcellular localization analysis revealed that these proteins were primarily located in mitochondria, mitochondrial membranes, lipid droplets, and other organellar membranes (Fig. 3G). Their molecular functions were mainly associated with ATPase-coupled transmembrane transporter activity, fatty acid ligase activity, CoA ligase activity, ATP binding, phospholipid transporter activity, and ion binding (Fig. 3H). These DEPs were involved in key biological processes such as regulation of cytochrome c release, glucose homeostasis, mitochondrial organization, CoA biosynthesis, fatty acid transport, tricarboxylic acid metabolism, and other mitochondrion-related functions (Fig. 3I). KEGG pathway analysis further demonstrated that the screened DEPs were significantly enriched in the citrate cycle (TCA cycle), fatty acid biosynthesis, ferroptosis, fatty acid degradation, and mitophagy (Fig. 3J).

Proteomic (PRO) analysis identified significant differential expression of two key proteins: OPA1, which is essential for mitochondrial function, and ACSL4, a central regulator of ferroptosis. Their expressions and roles were validated as followed through immunofluorescence, qPCR, PRM, molecular docking, phenotypic assays, and inhibitor experiments.

### QT ameliorates the abnormal expression of OPA1 and ACSL4 induced by AFB_1_

**Fig. 4:**
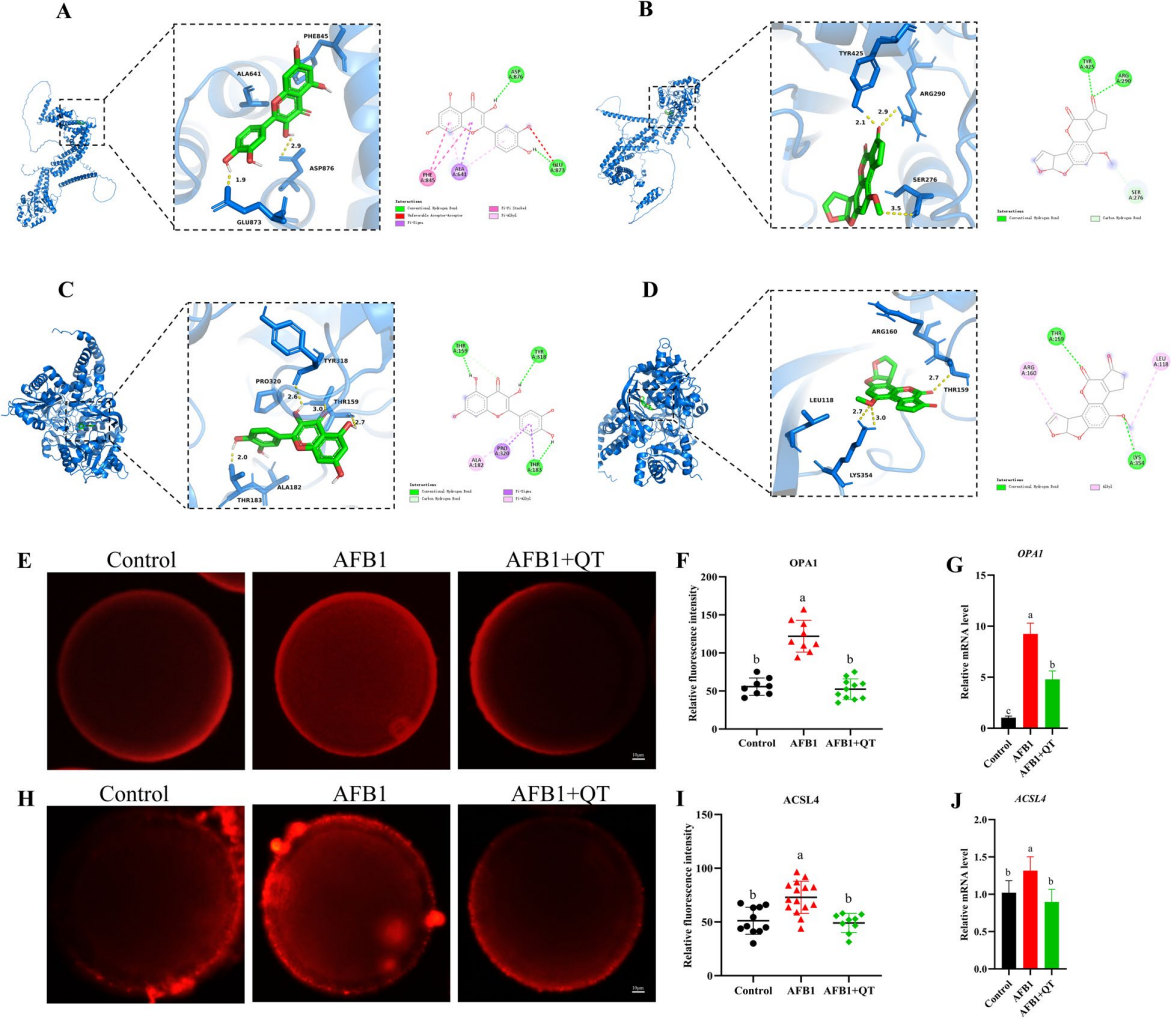
QT ameliorates the abnormal expression of OPA1 and ACSL4 proteins induced by AFB_1_. **A** AFB_1_ with OPA1. **B** QT with OPA1. **C** AFB_1_ with ACSL4. **D** QT with ACSL4. **A**–**D** For each panel, the left image shows the overall docking conformation, the middle image provides a magnified view of the binding site, and the right image is a two-dimensional schematic of the interaction. **E** and **F** Immunofluorescence staining and Relative fluorescence intensity of OPA1 in sheep oocyte. Scale bar: 10 μm. *n* = 30. **G ***OPA1* mRNA levels in the control, AFB_1_, and AFB_1_ + QT co-treatment groups. **H** Immunofluorescence staining of ACSL4 in sheep oocytes, Scale bar: 10 μm. **I** Relative fluorescence intensity values of ACSL4 in sheep oocytes. *n* = 30. **J ***ACSL4* mRNA levels in the control, AFB_1_, and AFB_1_ + QT co-treatment groups. ^a−c^Values with different superscripts indicate statistical significance (*P* < 0.05)

Molecular docking revealed that QT exhibited stronger binding affinity to both OPA1 and ACSL4 than AFB_1_. The docking score of QT with OPA1 was –8.166 kcal/mol, forming hydrogen bonds with ASP876 and GLU873, as well as π–π and π–alkyl interactions with PHE845 and ALA641 (Fig. 4A). In comparison, AFB_1_ binding to OPA1 yielded a score of −6.937 kcal/mol, involving hydrogen bonds with TYR425, ARG290, and SER276 (Fig. 4B). For ACSL4, QT achieved a docking score of −7.841 kcal/mol, engaging residues THR159, TYR318, and THR183 through hydrogen or carbon–hydrogen bonds, and ALA182, PRO320, and THR183 via π–π and π–alkyl interactions (Fig. 4C). AFB_1_ docking with ACSL4 resulted in a score of −7.72 kcal/mol, forming hydrogen bonds with THR159 and LYS354 and π–alkyl interactions with ARG160 and LEU118 (Fig. 4D).

Consistent with proteomic findings, AFB_1_ treatment significantly increased both protein fluorescence intensity and mRNA expression of OPA1 and ACSL4. However, QT co-treatment effectively reversed these alterations, restoring their expression to near-normal levels (*P* < 0.05, Fig. 4E–J). These results suggest that QT may exert its protective effect by competitively inhibiting AFB_1_ binding to OPA1 and ACSL4, thereby attenuating AFB_1_-induced dysregulation of these proteins. These results demonstrate that OPA1 and ACSL4 are not only responsive to AFB_1_ but also serve as direct targets of QT, thereby establishing a foundation for investigating their specific mechanisms of action at the cellular level.

### QT restores mitochondrial function and energy metabolism by correcting abnormal OPA1 expression

Proteomic analysis revealed a significant increase in OPA1 expression in AFB_1_-exposed ovine oocytes compared to the control group. This upregulation was consistently observed by immunofluorescence staining (Fig. 4E and F), qPCR (Fig. 4G), and PRM, which quantified a 1.82-fold elevation in OPA1 levels in the AFB_1_ group (Fig. 6A, H and I). In contrast, co-treatment with QT reduced OPA1 expression by 1.59-fold in the AFB_1_ + QT group (Fig. 6A, H and I), indicating that QT counteracts AFB_1_-induced OPA1 overexpression.

**Fig. 5:**
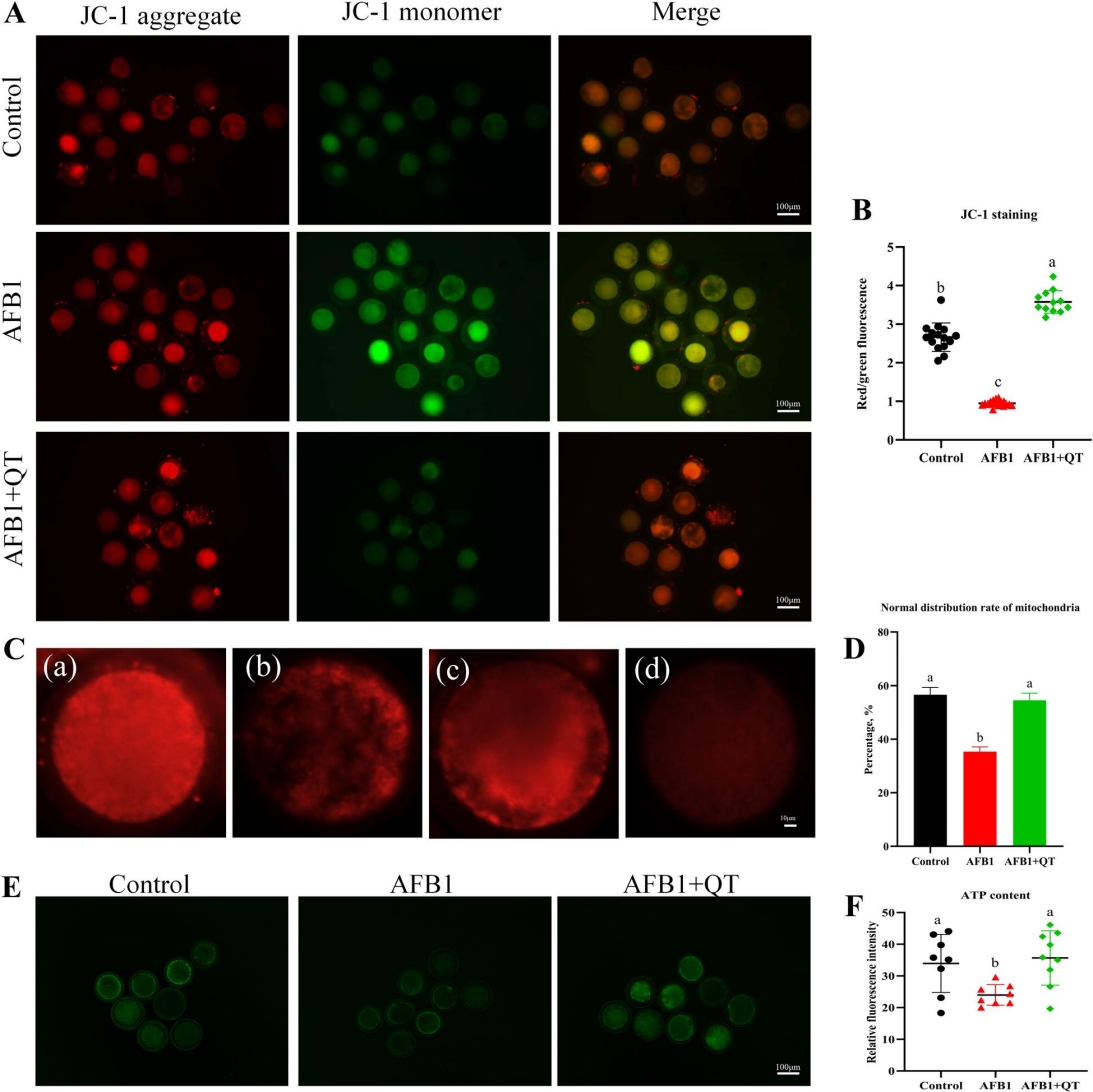
QT ameliorates mitochondrial function and energy metabolism by rescuing aberrant OPA1 expression. **A** Representative fluorescence images of mitochondrial membrane potential (ΔΨm) in the control, AFB_1_, and AFB_1_ + QT groups. Scale bar: 100 μm. **B** Quantification of mitochondrial membrane potential. *n* = 60. **C** Representative images depicting mitochondrial distribution patterns: (a) normal, homogeneous distribution; (b) aggregated distribution; (c) intermediate distribution; (d) distribution with low mitochondrial signal intensity. Scale bar: 10 μm. **D** Proportion of oocytes exhibiting normal mitochondrial distribution in the control, AFB_1_, and AFB_1_ + QT groups. *n* = 30. **E** Fluorescence-based quantification of ATP levels. Scale bar: 100 μm. **F** Quantitative analysis of ATP content.* n* = 45. ^a−c^Values with different superscripts indicate statistical significance (*P* < 0.05). Data are expressed as mean ± standard error of the mean

To evaluate the impact of AFB_1_-induced OPA1 upregulation on mitochondrial function, we assessed the mitochondrial membrane potential (ΔΨm) using JC-1 staining. The red/green fluorescence ratio was significantly lower in AFB_1_-treated oocytes than in controls. Co-treatment with QT restored ΔΨm to a level comparable to the control group (*P* < 0.05, Fig. 5A and B), indicating that QT alleviates AFB_1_-induced mitochondrial damage.

Mitochondrial distribution, visualized by MitoTracker staining, was homogeneous in most control and AFB_1_ + QT oocytes (Fig. 5C). In contrast, AFB_1_-exposed oocytes displayed abnormal patterns, including cytoplasmic aggregation, peripheral loss, and reduced intensity. The proportion of oocytes with normal mitochondrial distribution decreased significantly from 56.58% ± 1.60% in controls to 35.33% ± 1.02% in the AFB_1_ group. This was restored to 54.51% ± 1.53% with QT treatment (*P* < 0.05, Fig. 5D). These results demonstrate that QT rescues AFB_1_-induced disruption of mitochondrial distribution in ovine oocytes.

**Fig. 6:**
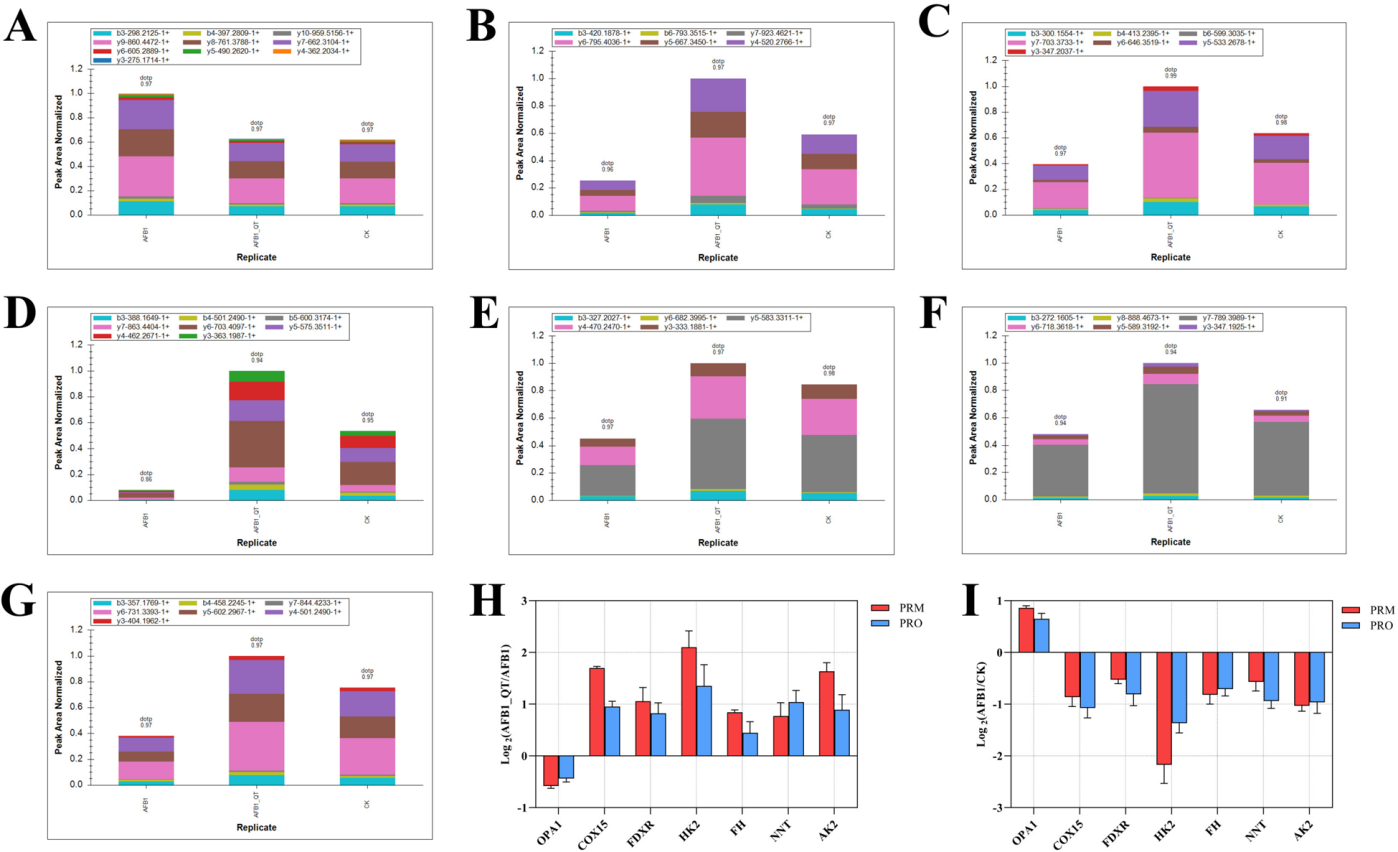
PRM-based verification of protein expression associated with mitochondrial function and energy metabolism. **A**–**G** Representative images showing fragment ion peak area distributions for unique peptides of OPA1 (VVVVGDQSAGK), COX15 (YQQFPEFK), FDXR (NVINTFTQTAR), HK2 (VCQIVSTR), FH (NVLHSAR), NNT (TVAELEAEK), and AK2 (APNVPAAEPVPESPK) proteins across control, AFB_1_, and AFB_1_ + QT groups. **H** and **I** Log_2_ fold changes for the seven proteins derived from PRO and PRM analyses, with the *y*-axis representing the Log_2_ ratio of unique peptide relative abundance between AFB_1_ + QT and AFB_1_ groups (**H**), and between AFB_1_ and control groups (**I**). Protein expression trends between the two treatment groups are expressed as mean values derived from PRO and PRM analyses, respectively. *n* = 45

PRM validation of proteomic data focused on AFB_1_-induced OPA1 upregulation showed that AFB_1_ significantly suppressed COX15 and FDXR expression by 1.84-fold and 1.52-fold, respectively. These proteins are essential for mitochondrial electron transport and antioxidant defense. QT co-treatment restored their expression to control levels (Fig. 6B and C, H and I), indicating that QT rescues mitochondrial function and antioxidant capacity by counteracting AFB_1_-induced OPA1 dysregulation.

Given the close relationship between mitochondrial function and energy metabolism, we next assessed ATP levels in oocytes using a mitochondrial ATP-specific fluorescent probe. AFB_1_-exposed oocytes showed significantly lower ATP levels than controls, an effect that was reversed by QT co-treatment, restoring ATP to baseline levels (Fig. 5E and F). Subsequent PRM validation guided by proteomic data indicated that AFB_1_-induced OPA1 upregulation was accompanied by significant downregulation of key proteins involved in glycolysis (HK2, decreased 2.78-fold), the tricarboxylic acid cycle (FH, decreased 1.75-fold), oxidative phosphorylation (NNT, decreased 1.5-fold), and energy homeostasis (AK2, decreased 2.04-fold). These changes collectively contributed to reduced ATP production. QT treatment restored the expression of these proteins to control levels (Fig. 6D–G, H and I), indicating that QT mitigates AFB_1_-induced energy metabolism disruption by normalizing OPA1 expression in ovine oocytes. The aberrant upregulation of OPA1 directly leads to a decrease in mitochondrial membrane potential, abnormal mitochondrial distribution, impaired ATP synthesis, and abnormal expression of related proteins, which constitutes the key organellar basis for AFB_1_-induced cellular stress and susceptibility to ferroptosis.

### QT inhibits ACSL4-dependent lipid peroxidation ferroptosis by correcting abnormal ACSL4 expression

Proteomic analysis, immunofluorescence staining, and qPCR consistently showed that AFB_1_ treatment significantly increased both ACSL4 protein and mRNA expression (Figs. 4H–J and 8H–J). PRM-based targeted proteomics further confirmed a 1.6-fold upregulation of ACSL4 protein after AFB_1_ exposure. In contrast, co-treatment with QT reduced ACSL4 expression by 1.55-fold compared to the AFB_1_ group (Fig. 8A, H and I), suggesting that QT protects ovine oocytes by inhibiting the ACSL4-mediated lipid peroxidation pathway.

**Fig. 7:**
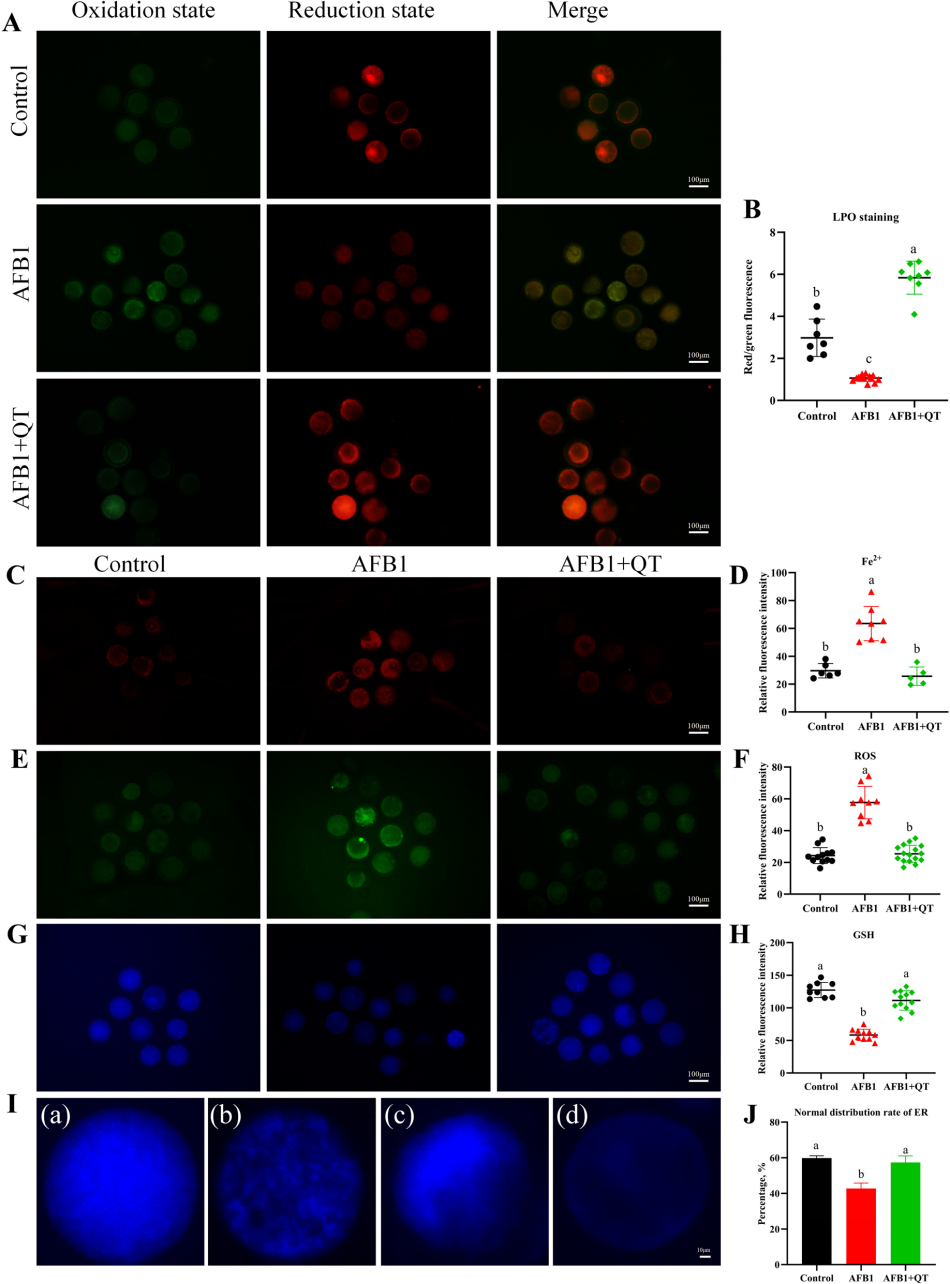
ACSL4-dependent lipid peroxidation cascade induces ferroptosis initiation. **A**–**H** LPO fluorescence signals (**A**), relative fluorescence intensity of LPO (**B**), Fe^2+^ fluorescence signals (**C**), Relative fluorescence intensity of Fe^2+^ (**D**), ROS fluorescence signals (**E**), Relative fluorescence intensity of ROS (**F**), GSH fluorescence signals (**G**), Relative fluorescence intensity of ROS (**H**) in ovine oocytes across various treatment groups. Scale bar: 100 μm. *n* = 45. **I** Representative images depicting patterns of endoplasmic reticulum distribution. Scale bar: 10 μm. (a) Regular distribution. (b) Clustered distribution. (c) Semi-peripheral distribution. *n* = 45. (d) Distribution with minimal endoplasmic reticulum signal. **J** Proportion of normal endoplasmic reticulum distribution observed in the control, AFB_1_, and AFB_1_ + QT groups. *n* = 30. ^a^^−^^c^Values with different superscripts indicate statistical significance (*P* < 0.05). Data are expressed as mean ± standard error of the mean

To determine whether QT protects oocytes by suppressing ACSL4-dependent lipid peroxidation and reducing Fe^2+^ accumulation, lipid peroxidation was assessed using the BODIPY 581/591 C11 probe. Oocytes from the control and AFB_1_ + QT groups exhibited predominantly red fluorescence, whereas AFB_1_ exposure significantly reduced red fluorescence and increased green fluorescence, leading to a lower red-to-green ratio compared with the control and AFB_1_ + QT groups (*P* < 0.05, Fig. 7A and B). In addition, we also measured intracellular Fe^2+^ levels. AFB_1_-treated oocytes showed stronger red fluorescence, indicating Fe^2+^ accumulation, while QT co-treatment restored fluorescence intensity to baseline (*P* < 0.05, Fig. 7C and D). These results demonstrate that QT alleviates AFB_1_-induced ferroptosis in oocytes by normalizing ACSL4 expression, reducing Fe^2+^ accumulation, and inhibiting polyunsaturated fatty acid peroxidation.

To assess oxidative stress status during ferroptosis and the regulatory effects of AFB_1_ and QT, we measured intracellular ROS levels and glutathione (GSH) content. AFB_1_ exposure significantly increased ROS accumulation and depleted GSH, indicating severe oxidative stress and compromised antioxidant defense (*P* < 0.05, Fig. 7E–H,). In contrast, co-treatment with QT effectively reduced ROS levels and restored GSH content, demonstrating its ability to alleviate AFB_1_-induced oxidative damage (*P* < 0.05, Fig. 7E–H).

To evaluate the effect of QT on AFB_1_-induced endoplasmic reticulum (ER) stress, ovine oocytes were stained with ER-Tracker Blue. As shown in Fig. 7I and J, the proportion of oocytes displaying normal ER distribution was significantly lower in the AFB_1_ group (42.73% ± 1.77%) than in the control (59.79% ± 0.76%) and AFB_1_ + QT groups (*P* < 0.05, 57.37% ± 2.1%), indicating that QT ameliorates AFB_1_-induced ER stress. The aberrant upregulation of ACSL4 and the consequent lipid peroxidation are direct contributors to AFB_1_-induced ferroptosis in oocytes. QT comprehensively blocks this process, consistent with its role in modulating ACSL4 expression.

**Fig. 8:**
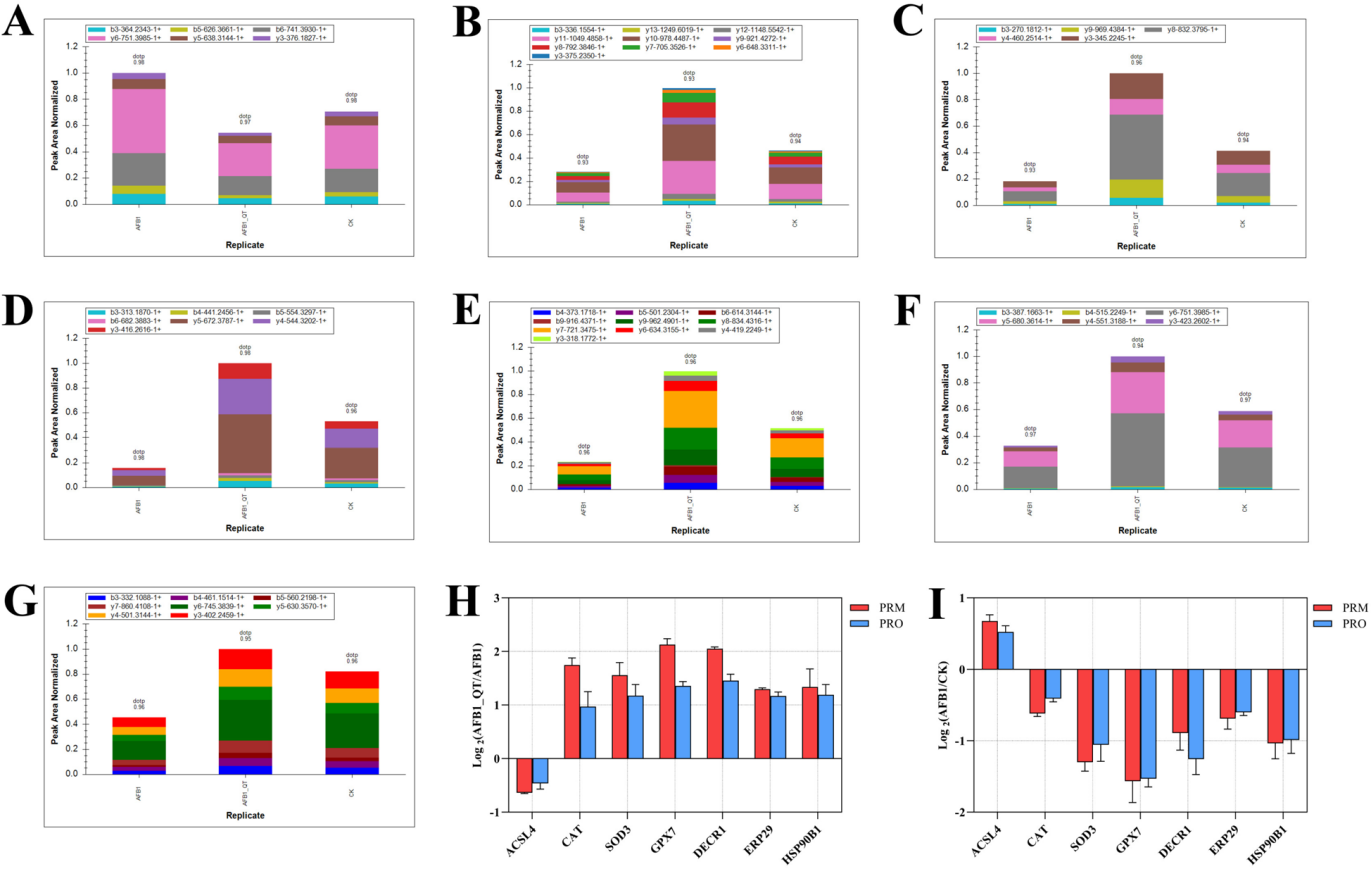
PRM validation of protein expression associated with lipid peroxidation, ferroptosis, and endoplasmic reticulum stress. **A–G** Representative images showing fragment ion peak area distributions for unique peptides of ACSL4 (HIIYVDNK), CAT (YQQFPEFK), SOD3 (AVVVHAGEDDLGR), GPX7 (ALQQLQR), DECR1 (ATAEQISSQTGNK), ERP29 (WAEQYLK), and HSP90B1 (TDDEVVQR) proteins across control, AFB_1_, and AFB_1_ + QT groups. **H** and **I** Log_2_ fold changes for the seven proteins derived from proteomic (PRO) and PRM analyses, with the *y*-axis representing the Log_2_ ratio of unique peptide relative abundance between AFB_1_ + QT and AFB_1_ groups (**H**), and between AFB_1_ and control groups (**I**). *n* = 45

Based on PRM validation of proteomic data, AFB_1_ exposure in ovine oocytes significantly down regulated the expression of key antioxidant enzymes: CAT (1.54-fold), SOD3 (2.27-fold), and GPX7 (2.94-fold) compared to the control group, thereby impairing cellular ROS-scavenging capacity (Fig. 8B–D, H and I). AFB_1_ also suppressed DECR1 expression by 2.19-fold, disrupting lipid homeostasis (Fig. 8E, H and I). Additionally, ERP29 and HSP90B1 were downregulated by 1.69-fold and 1.85-fold, respectively, leading to dysregulated protein folding and calcium homeostasis, and inducing endoplasmic reticulum stress (Fig. 8F and G, H and I). Co-treatment with QT effectively upregulated these proteins, restoring their expression to near-normal levels and counteracting AFB_1_-induced damage via enhanced antioxidant capacity, improved lipid and iron metabolism, and alleviated ER stress (Fig. 8A–I).

### QT protects against AFB_1_-induced iron-dependent non-canonical ferroptosis in oocytes via the OPA1/ACSL4 pathway

**Fig. 9:**
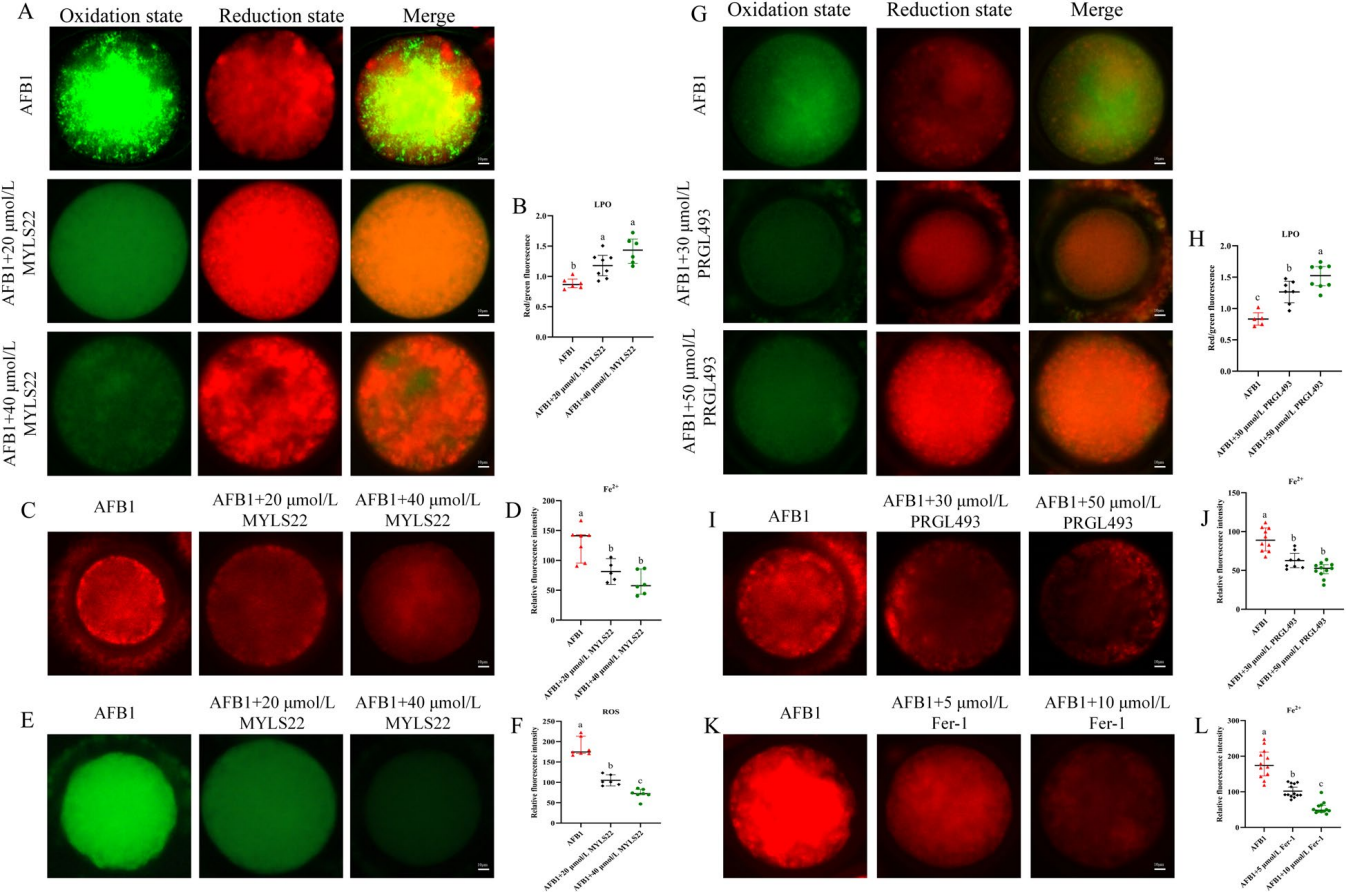
Determination of the working concentrations for MYLS22, PRGL493, and Ferrostatin-1. **A**–**F** Lipid peroxidation (LPO) results (**A**), relative fluorescence intensity of LPO (**B**), Fe^2+^ levels (**C**), relative fluorescence intensity of Fe^2+^ (**D**), ROS levels (**E**), and relative fluorescence intensity of ROS (**F**) in oocytes treated with 15 μmol/L AFB1 under graded concentrations of MYLS22. Scale bar: 10 μm. *n* = 30. **G**–**J** Lipid peroxidation (LPO) results (**G**), relative fluorescence intensity of LPO (**H**), Fe^2+^ levels (**I**), and relative fluorescence intensity of Fe^2+^ (**J**) in oocytes treated with 15 μmol/L AFB1 under graded concentrations of PRGL493. Scale bar: 10 μm. *n* = 30. **K** and **L** The concentrations Fe^2+^ (**K**) and the relative fluorescence intensity of Fe^2+^ (**L**) in oocytes treated with 15 μmol/L AFB_1_ under different amounts of Ferrostatin-1 (Fer-1). Scale bar: 10 μm. *n* = 30. ^a−c^Values with different superscripts indicate statistical significance (*P* < 0.05)

To definitively assess the roles of OPA1 and ACSL4 in QT-mediated protection against AFB_1_-induced ferroptosis, we employed MYLS22 (an OPA1 inhibitor), PRGL493 (an ACSL4 inhibitor), and Ferrostatin-1 (Fer-1, a ferroptosis inhibitor). Treatment with 20 μmol/L MYLS22 significantly reduced AFB_1_-induced increases in LPO, Fe^2+^, and ROS levels (Fig. 9A–F). Similarly, 30 μmol/L PRGL493 markedly attenuated AFB_1_-triggered LPO and Fe^2+^ accumulation (Fig. 9G–J). Administration of 5 μmol/L Fer-1 also significantly decreased Fe^2+^ levels (Fig. 9K and L). Based on these results, the working concentrations for subsequent experiments were set at 20 μmol/L for MYLS22, 30 μmol/L for PRGL493, and 5 μmol/L for Fer-1.

**Fig. 10:**
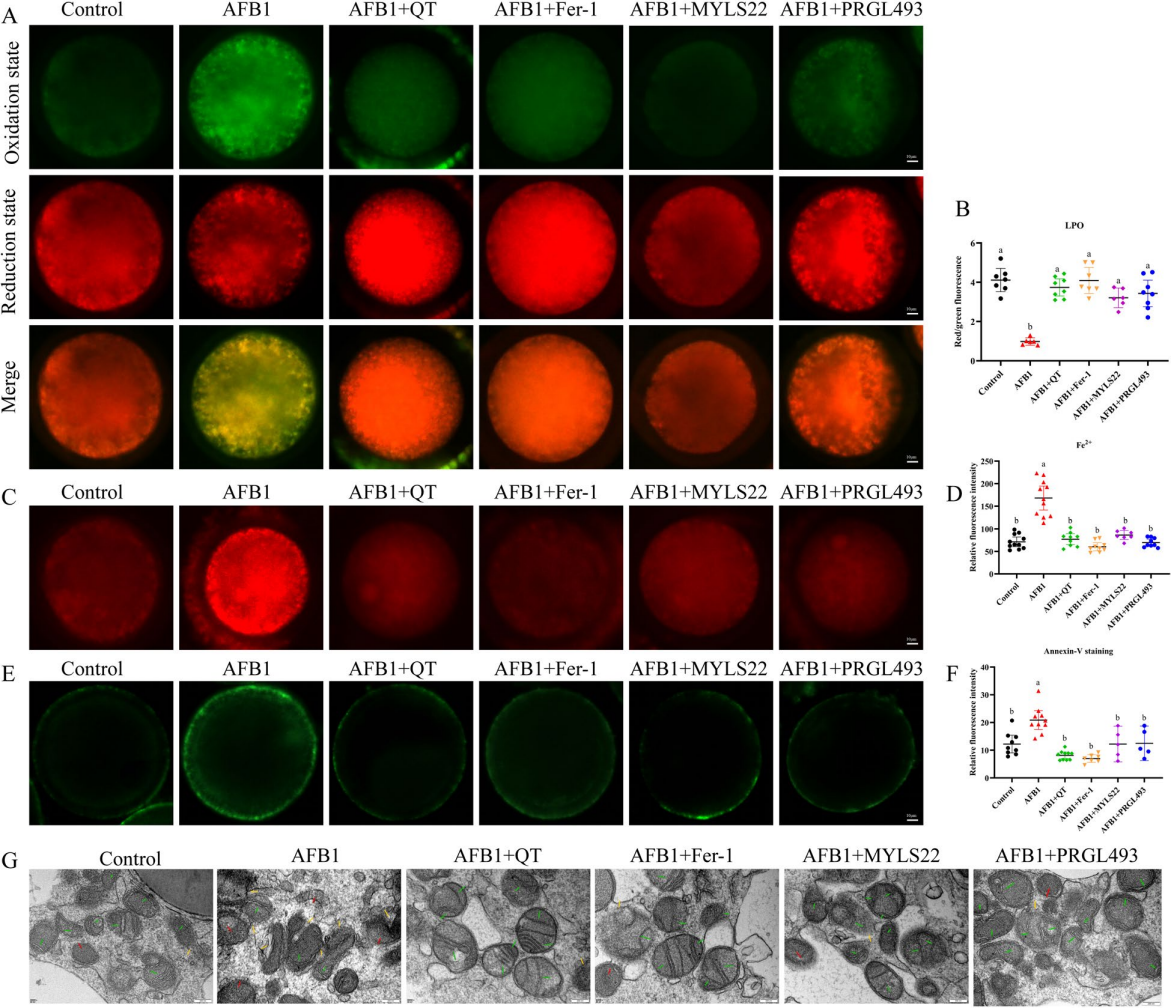
Inhibition of AFB_1_-induced aberrant OPA1 and ACSL4 expression alleviates ferroptosis in sheep oocytes. **A**–**F** LPO fluorescence signals (**A**), comparative fluorescence intensity of LPO (**B**), fluorescence signals of Fe^2+^ (**C**), fluorescence intensity of Fe^2+^ (**D**), fluorescence signals of Annexin-V (**E**), and fluorescence intensity of Annexin-V (**F**) in ovine oocytes within various treatment groups. Scale bar: 10 μm. **G** Examination of mitochondrial ultrastructure in ovine oocytes from each category. Green arrows indicate mitochondrial cristae; yellow arrows highlight abnormal structures of the mitochondrial membrane; red arrows denote the absence of mitochondrial cristae. Scale bar: 200 nm. ^a,b^Values with different superscripts indicate statistical significance (*P* < 0.05)

To determine whether the protective effect of QT against AFB_1_-induced oocyte damage depends on OPA1 and ACSL4 activity, COCs were co-cultured with AFB_1_ in the presence of the OPA1 inhibitor MYLS22 or the ACSL4 inhibitor PRGL493 for 23 h. LPO, Fe^2+^ levels, and apoptosis were then assessed in sheep oocytes across the control, AFB_1_, AFB_1_ + QT, and Fer-1 groups. As shown in Fig. 10A–D, both MYLS22 and PRGL493 abolished AFB_1_-induced LPO and reduced Fe^2+^ accumulation. The LPO and Fe^2+^ intensities in inhibitor-treated groups did not differ significantly from those in the control, AFB_1_ + QT, and Fer-1 groups (*P* > 0.05). Moreover, MYLS22 and PRGL493 suppressed AFB_1_-triggered oocyte apoptosis, with Annexin-V staining intensities similar to the control, AFB_1_ + QT, and Fer-1 groups (*P* > 0.05, Fig. 10E and F). Transmission electron microscopy further revealed that oocytes exposed to 15 μmol/L AFB_1_ exhibited pronounced mitochondrial cristae loss and membrane disruption. In contrast, supplementation with QT, Fer-1, MYLS22, or PRGL493 restored normal ultrastructure, characterized by well-defined cristae and intact double membranes (Fig. 10G). Together, these results demonstrate that inhibiting OPA1 or ACSL4 activity attenuates AFB_1_-induced lipid peroxidation and Fe^2+^ accumulation, and confirm that QT protects ovine oocytes from AFB_1_-triggered ferroptosis by suppressing OPA1/ACSL4-mediated lipid peroxidation.

The canonical ferroptosis is primarily regulated by glutathione peroxidase 4 (GPX4) and transferrin receptor 1 (TFRC/TFR1), which the decrease in GPX4 activity and the increase in TFRC expression lead to an increase in ROS and the accumulation of intracellular Fe^2+^, resulting in ferroptosis [34]. However, in our study, the proteomic (PRO) and PRM analyses both identified that the expressions of GPX4 and TFRC proteins did not follow the canonical ferroptosis pathway (Fig. S1A–D). The results of proteomic (PRO) analysis showed that the expression of GPX4 was not significantly different in each group, while TFRC was down-regulated in the AFB_1_ group. The results of PRM for quantitative detection of GPX4 and TFRC showed that the expression of GPX4 was not significantly different in each group, and the expression of TFRC in the AFB_1_ group was abnormally down-regulated by 3.7 times compared to the control group and 4.85 times compared to the AFB_1_ + QT group, which are consistent with the proteomic results (Fig. S1A–D). Thus, we suggest that QT ameliorates AFB_1_-induced iron-dependent cell death in ovine oocytes via a non-canonical ferroptosis pathway.

**Fig. 11:**
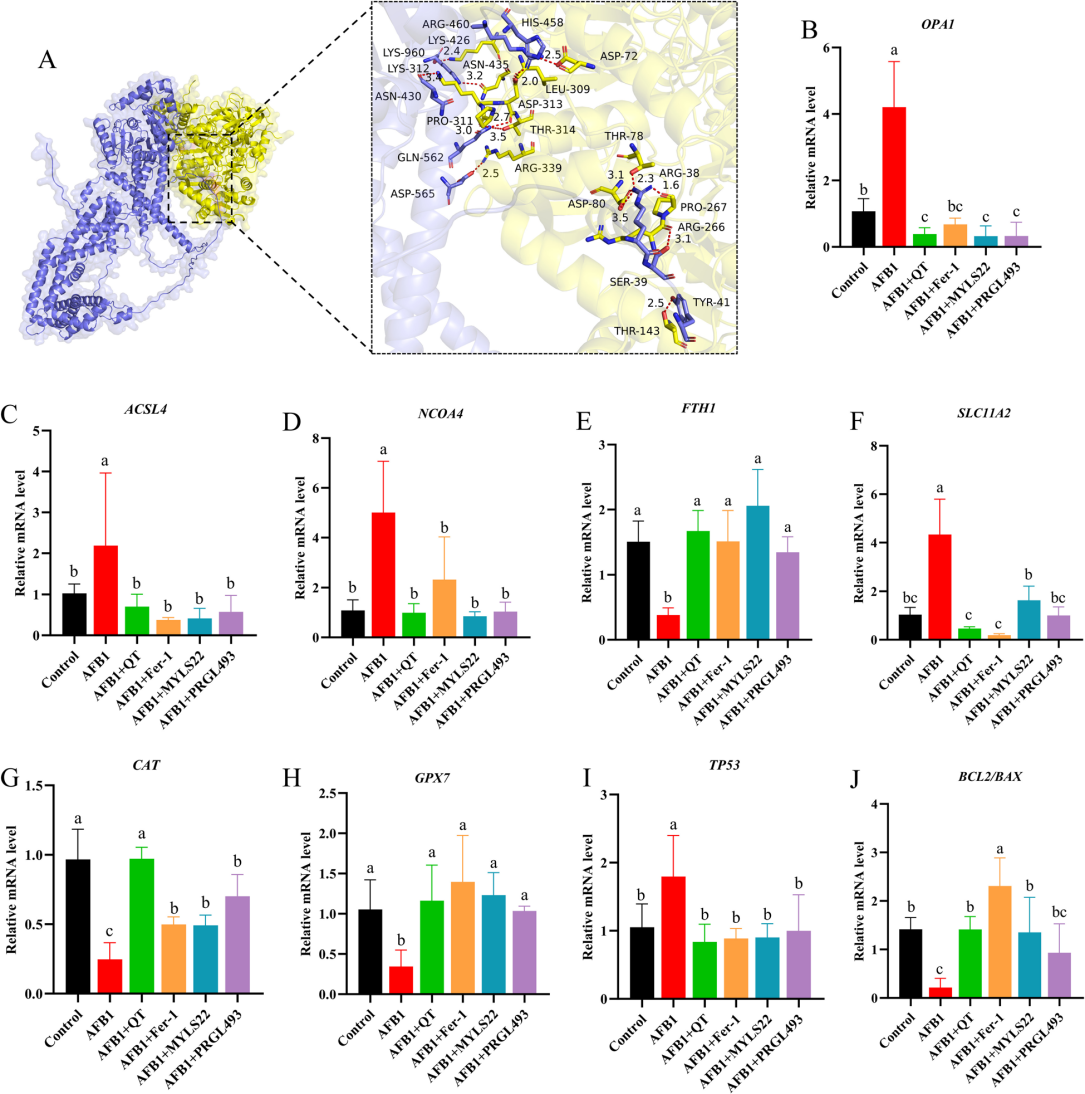
QT suppresses AFB_1_-induced iron-dependent non-canonical ferroptosis in oocytes via the OPA1/ACSL4 pathway. **A** Molecular docking analysis results for the OPA1 and ACSL4 interaction. The left panel shows the overall docking conformation; the right panel provides a magnified view of the binding site. Red dashed lines indicate hydrogen bonds, with adjacent numbers denoting bond lengths. Effective hydrogen bond lengths fall within the 1.5–3.5 Å range; within this interval, a shorter bond length indicates greater amino acid residue criticality. Amino acid residues connected by the red dashed lines represent interaction sites; yellow indicates residues from the OPA1 protein, purple indicates residues from the ACSL4 protein. **B**–**J** *OPA1*, *ACSL4*, *NCOA4*, *FTH1*, *SLC11A2*,* CAT*,*GPX7*,* TP53 *and* BCL2/BAX* mRNA levels across different treatment groups

Molecular docking between OPA1 and ACSL4 was performed using HDOCK to assess their potential interaction. The predicted complex structure shows OPA1 (purple) and ACSL4 (yellow) forming a stable interface, with binding stabilized by multiple hydrogen bonds (red dashed lines in Fig. 11A). Key residues in the OPA1 domain (ARG460, HIS458, LYS960, ASN430, GLN562, ASP565, TYR41, SER39, and ARG38) interact with residues in the ACSL4 domain (ASP72, LEU309, LYS462, ASN435, PRO311, ASP313, THR314, ARG339, ASP80, THR78, PRO267, ARG266, and THR143). A docking score of –292.97 indicates strong binding affinity between the two proteins. To functionally validate this interaction, we examined the regulatory relationship between OPA1 and ACSL4 using specific inhibitors. When AFB_1_-exposed oocytes were treated with the ACSL4 inhibitor PRGL493, *OPA1* mRNA levels decreased significantly compared to the AFB_1_-only group (*P* < 0.05) and did not differ significantly from the control, AFB_1_ + QT, AFB_1_ + Fer-1, or AFB_1_ + MYLS22 groups (*P* > 0.05, Fig. 11B). Conversely, treatment with the OPA1 inhibitor MYLS22 significantly reduced *ACSL4* mRNA expression relative to the AFB_1_ group, reaching levels comparable to the control and other treatment groups (*P* > 0.05, Fig. 11C). These results confirm that OPA1 and ACSL4 not only physically interact but also mutually upregulate each other’s expression.

To assess key iron metabolism genes, we found that AFB_1_ exposure significantly upregulated *NCOA4* (involved in ferritin degradation) and *SLC11A2* (an iron transporter), while suppressing *FTH1 *(an iron-storage protein) and its gene expression (Fig. S1E and F). These effects were reversed by MYLS22 or PRGL493, which reduced *SLC11A2* and *NCOA4* expression and elevated *FTH1* levels (*P* < 0.05, Fig. 11D–F), indicating that inhibition of OPA1 or ACSL4 normalizes iron handling and limits Fe^2+^ accumulation. Furthermore, both inhibitors enhanced the expression of antioxidant genes (*CAT* and *GPX7*), increased the *BCL2/BAX* ratio, and suppressed *TP53* expression in AFB_1_-treated oocytes (*P* < 0.05, Fig. 11G–J), indicating improved antioxidant capacity and reduced apoptosis. Together, these findings demonstrate that inhibition of OPA1 or ACSL4 alleviates AFB_1_-induced ferroptosis and cellular injury, and confirm that QT protects oocytes by modulating the OPA1/ACSL4/NCOA4 pathway in a non-canonical ferroptosis mechanism. Inhibitor assays and protein interaction analyses established the central and synergistic roles of OPA1 and ACSL4 in the AFB_1_ pathway, revealing a downstream, non-classical mechanism of iron metabolism regulation via NCOA4. These findings comprehensively elucidate the molecular mechanism whereby QT inhibits AFB_1_-induced non-canonical ferroptosis in ovine oocytes by targeting the OPA1/ACSL4/NCOA4 axis.

## Discussion

Aflatoxin B_1_ is a widespread environmental contaminant that jeopardizes reproductive health in humans and animals [9]. In sheep oocytes, AFB_1_ exposure reduces the meiosis II (MII) maturation rate and induces oxidative stress and apoptosis [14]. Our data further show that AFB_1_ internalization intensifies oxidative damage and apoptosis, triggers lipid peroxidation and ferroptosis, and ultimately leads to oocyte maturation failure. As no effective treatment is currently available to counteract AFB_1_-induced oocyte injury, identifying potential protective agents is essential. Quercetin (QT), a naturally occurring flavonoid, possesses a broad spectrum of biological activities, including antioxidant, anti-inflammatory, immunomodulatory, and cytoprotective properties [24]. Previous studies have shown that QT improves the in vitro maturation and early embryonic development of human and aged mouse oocytes by reducing mitochondrial reactive oxygen species, suppressing apoptosis, and enhancing autophagic activity [27]. However, whether QT can protect sheep oocytes against AFB_1_ injury remains unknown. In this study, we first evaluated the effects of different concentrations of AFB_1_ and QT on the MII maturation rate and early apoptosis in sheep oocytes. We then applied 4D-FastDIA quantitative proteomics to compare protein expression profiles across control, AFB_1_-treated, and AFB_1_ + QT co-treated groups. Key findings were validated using PRM-based targeted proteomics, qPCR, and inhibitor assays. Our results demonstrate that QT significantly attenuates AFB_1_-induced damage in sheep oocytes by improving MII maturation and mitochondrial function, while reducing early apoptosis, oxidative stress, lipid peroxidation, iron overload, and endoplasmic reticulum stress.

During in vitro maturation, AFB_1_ exposure impaired both nuclear and cytoplasmic maturation of oocytes in a dose dependent manner. Consistent with previous findings that blastocyst formation rates decreased with increasing AFB_1_ concentrations and were completely suppressed at 400 and 4,000 µg/L [12], our preliminary experiments confirmed a dose-dependent inhibition of polar body extrusion. Prior studies have shown that AFB_1_ downregulates the anti-apoptotic gene *BCL2* and DNA methyltransferase DNMT3b, upregulates pro-apoptotic genes *BAX* and Caspase3, and induces mitochondrial dysfunction, oxidative stress, and reduced MII maturation rates in sheep oocytes [14]. In agreement, we observed that 15 and 30 μmol/L AFB_1_ significantly suppressed polar body extrusion, elevated *TP53* expression, and decreased the *BCL2/BAX* ratio. Based on these results, 15 μmol/L AFB_1_ was selected for subsequent experiments. Importantly, we demonstrated that QT restores the developmental competence of AFB_1_-exposed oocytes, suppresses *TP53* expression, and enhances the *BCL2/BAX* ratio, thereby attenuating AFB_1_-induced early apoptosis. These findings indicate that QT protects against AFB_1_-induced oocyte damage by promoting nuclear maturation and inhibiting apoptotic pathways.

Functional enrichment analysis was performed on the DEPs between the AFB_1_ + QT and AFB_1_ groups, which were associated with mitochondrial function, lipid metabolism, ATP energy metabolism, cellular stress, endoplasmic reticulum, and membrane structures. This analysis revealed significant enrichment in key pathways, including the citrate cycle (TCA cycle), fatty acid biosynthesis, ferroptosis, fatty acid degradation, and mitophagy. Among these DEPs, the mitochondrial protein OPA1 and the ferroptosis-related protein ACSL4 were markedly upregulated in the AFB_1_ group. Molecular docking further confirmed that both QT and AFB_1_ can directly bind to OPA1 and ACSL4, respectively.

Mitochondria are the primary energy source in eukaryotic cells, sustaining cellular activities through continuous ATP production. External stimuli can disrupt mitochondrial homeostasis, leading to mitochondrial stress [35]. OPA1, a dynamin-like GTPase located in the mitochondrial inner membrane, is essential for mitochondrial fusion, electron transport chain supercomplex assembly, cristae remodeling, mitochondrial DNA maintenance, and energy metabolism [29]. In this study, AFB_1_ exposure triggered a 1.82-fold upregulation of OPA1 expression, which subsequently reduced the mitochondrial membrane potential (ΔΨm) and caused abnormal mitochondrial distribution. Aberrant OPA1 upregulation has been shown to impair mitochondrial fusion, disrupt mitochondrial structure and function, and promote ferroptosis [32, 36, 37]. We found that AFB1-induced OPA1 overexpression destabilized ΔΨm, leading to mitochondrial aggregation and peripheral loss in place of the normal homogeneous cytoplasmic distribution. This was accompanied by suppressed expression of key electron transport chain and antioxidant defense proteins, ultimately inducing oocyte ferroptosis, a finding consistent with reports by Liang et al. [32] and Weng et al. [36]. Previous studies indicate that oocyte-specific knockout of COX15 disrupts Fe^2+^ and ROS homeostasis, promoting mitochondrial dysfunction and increasing susceptibility to ferroptosis [38]. Similarly, FDXR deficiency induces mitochondrial iron overload via iron regulatory protein 2 (IRP2) [39], which aligns with our observation that AFB1-induced OPA1 upregulation suppressed both COX15 and FDXR expression. These alterations further impaired ATP production and disrupted overall energy metabolism in oocytes. Downregulation of key metabolic enzymes, including HK2, FH, NNT, and AK2, has been linked to diminished glycolytic flux, interference with the TCA cycle, reduced NADPH availability, and disrupted iron-sulfur cluster homeostasis, collectively promoting ferroptosis and impairing cellular development [40–43] and corroborating our findings. Notably, co-treatment with QT significantly alleviated AFB1-induced mitochondrial dysfunction and metabolic impairment. Consistent with studies showing that QT enhances mitochondrial membrane potential, glutathione levels, and ATP production in AFB1-exposed hepatocytes [44, 45], we observed that QT intervention normalized OPA1 expression, restored ΔΨm and mitochondrial distribution, and rescued the expression of electron transport chain and antioxidant proteins. These results suggest that QT counteracts ferroptosis, by modulating OPA1 to restore mitochondrial integrity and function.

The acyl-CoA synthetase long-chain (ACSL) family enzymes "activate" polyunsaturated fatty acids (PUFAs) and monounsaturated fatty acids (MUFAs) to generate the corresponding acyl-CoA esters (PUFA-CoA and MUFA-CoA). These products can then be incorporated into membrane phospholipids, triacylglycerols (TAG), or other lipid species [46]. Specifically, ACSL4-mediated metabolism of long-chain PUFAs, such as arachidonic acid (AA) and adrenic acid (AdA), promotes cellular susceptibility to ferroptosis. This process entails the esterification of PUFAs into their CoA esters (PUFA-CoA), which are subsequently integrated into membrane phospholipids, generating substrates for lipid peroxidation [30, 31]. Aberrant upregulation of ACSL4 increases the PUFA content within membrane phospholipids, thereby providing ample substrate for peroxidation [15, 16]. Existing evidence confirms that AFB_1_ induces lipid peroxidation in human and mouse hepatocytes [47, 48]. Our investigation of AFB_1_-treated ovine oocytes revealed that ACSL4 upregulation significantly elevated lipid peroxidation. Critically, lipid peroxidation products can disrupt iron-regulatory proteins or promote iron release, increasing free Fe^2+^ levels. This Fe^2+^ then fuels further lipid peroxidation via the Fenton reaction, establishing a self-perpetuating cycle [15, 16], which aligns with our observation of significantly elevated intracellular Fe^2+^ following AFB_1_ exposure. Fe^2+^ accumulation-triggered PUFA peroxidation is typically accompanied by oxidative stress, characterized by elevated ROS, glutathione (GSH) depletion, and endoplasmic reticulum stress [17, 49], consistent with our findings in AFB_1_-treated oocytes. CAT and SOD3 suppress lipid peroxidation by scavenging reactive oxygen species, reducing ferroptosis risk [9, 47]. GPX7, an ER-localized peroxidase, clears lipid peroxides and complements GPX4; its loss promotes ferroptosis [50]. ERP29 (Endoplasmic Reticulum Protein 29) regulates ER protein folding and calcium homeostasis. ER stress and ferroptosis are closely interconnected; sustained ER stress promotes lipid peroxidation and ferroptosis [51]. DECR1 (2,4-dienoyl-CoA reductase 1) is involved in polyunsaturated fatty acid β-oxidation. Downregulated DECR1 causes aberrant arachidonic acid metabolism and upregulates ACSL4, increasing cellular susceptibility to ferroptosis [52]. Supporting this, Elmorsy et al. [47] demonstrated that AFB_1_ suppresses key antioxidant components, including CAT, SOD, NRF2, and HO-1 gene expression, leading to increased ROS and lipid peroxidation. Our PRM validation, guided by proteomic data, confirmed significant downregulation of the antioxidant enzymes CAT, SOD3, and GPX7 in the AFB_1_ group, which severely compromised cellular ROS-scavenging capacity. Furthermore, Yang et al. [52] showed via multi-omics analysis that DECR1 knockdown induces ferroptosis, suppresses arachidonic acid (AA) production, upregulates ACSL4 expression, and downregulates the ferroptosis inhibitors GPX4 and SLC7A11. Our observation of a 2.19-fold downregulation of DECR1 concomitant with AFB_1_-induced ACSL4 upregulation is consistent with this report. Additionally, the downregulation of ERP29 and HSP90B1 expression likely contributed to endoplasmic reticulum stress in AFB_1_-treated oocytes, mirroring findings from Lu et al. [53] and Thaxton et al. [54]. Importantly, our results demonstrate that QT treatment effectively mitigates AFB_1_-induced lipid peroxidation and ferroptosis in ovine oocytes. Quercetin possesses potent antioxidant activity. Previous studies indicate that QT protects hepatocytes and mouse neurons from AFB_1_ injury by reducing ROS generation and lipid peroxidation while enhancing GSH levels and the activity of SOD and CAT [44, 55]. Similarly, quercetin and its derivative isorhamnetin inhibit ROS generation and cytotoxicity in AFB_1_-treated HepG2 cells. Oral quercetin administration in mice alleviates AFB_1_-induced toxicity by reducing serum lactate dehydrogenase, increasing hepatic GSH and superoxide dismutase activity, and attenuating lipid peroxidation in liver and kidney tissues [56]. Collectively, these data indicate that quercetin counteracts AFB_1_-induced lipid peroxidation and ferroptosis by inhibiting aberrant ACSL4 upregulation, enhancing the expression of antioxidant proteins CAT, SOD3, GPX7 and DECR1. Consequently, QT significantly reduces LPO, Fe^2+^ concentration, ROS levels, and endoplasmic reticulum stress, while elevating GSH expression.

The canonical ferroptosis pathway generally depends on GPX4 inactivation and TFRC upregulation, which together drive lipid peroxidation by suppressing the XCT–GSH–GPX4 axis and enhancing iron uptake [34]. In contrast to canonical ferroptosis, which depends on GPX4 inactivation, glutathione depletion, and TFRC-mediated iron uptake, non-canonical ferroptosis represents a distinct programmed cell death pathway that is similarly iron-dependent and involves lipid peroxidation but operates through alternative mechanisms. In this study, however, GPX4 and TFRC expression in AFB_1_-treated oocytes differed from this classical ferroptosis profile. Specifically, TFRC expression was significantly downregulated after AFB_1_ exposure, while GPX4 levels remained unchanged across groups. These findings suggest that AFB_1_ triggers ferroptosis independently of conventional GPX4 inactivation or TFRC-mediated iron uptake. We found that AFB_1_ exposure markedly increased mRNA levels of nuclear receptor coactivator 4 (*NCOA4*), promoting the degradation of ferritin heavy chain 1 (*FTH1*), as evidenced by significant reductions in both FTH1 immunofluorescence intensity and mRNA abundance. FTH1 converts and stores Fe^2+^ as Fe^3+^, thereby limiting free iron available for the Fenton reaction and suppressing lipid peroxidation [57]. *NCOA4* primarily promotes ferroptosis by mediating ferritin degradation; enhanced *NCOA4* activity leads to FTH1 breakdown and subsequent iron release [58]. In addition, AFB_1_ treatment significantly upregulated mRNA expression of solute carrier family 11 member 2 (SLC11A2), a key transmembrane iron transporter. Elevated *SLC11A2* expression increases cytosolic Fe^2+^ concentrations, expanding the labile iron pool (LIP) [59]. Thus, NCOA4-mediated iron release from FTH1 degradation, together with SLC11A2-driven iron influx, leads to excessive intracellular Fe^2+^ accumulation, initiating lipid peroxidation and ferroptosis via a mechanism distinct from GPX4 inactivation or TFRC upregulation. This model aligns with several non-canonical ferroptosis pathways reported in various cellular and pathological contexts. For example, Yang et al. [60] described a novel non-canonical ferroptosis inhibitor, YL-939, which binds prohibitin 2 and enhances ferritin expression, thereby reducing intracellular iron levels and ferroptosis susceptibility. Wu et al. [61] identified a phenazine derivative, compound 13 l, that acts as a non-canonical ferroptosis inhibitor by blocking NCOA4-mediated ferritinophagy, providing cellular protection. Moreover, He et al. [62] reported that hemin and chlorin e6 activate ferroptosis through a dual mechanism involving both classical GPX4 downregulation and non-canonical Fe^2+^ overload. The existence of such diverse pathways broadens our understanding of the regulatory network governing cell death and supports the development of specific therapeutic interventions. Importantly, QT co-treatment effectively normalized the aberrant expression of NCOA4 and SLC11A2 and enhanced FTH1 expression, leading to reduced intracellular free iron and restored iron homeostasis. This supports the conclusion that QT alleviates non-canonical ferroptosis by modulating iron metabolism, consistent with previous studies [60, 61].

To determine whether QT ameliorates AFB_1_-induced oocyte damage and non-canonical ferroptosis mediated by aberrant OPA1 and ACSL4 upregulation, we employed the OPA1 inhibitor MYLS22, the ACSL4 inhibitor PRGL493, and the broad-spectrum ferroptosis inhibitor Ferrostatin-1 (Fer-1) in validation experiments. Our results show that both MYLS22 and PRGL493 significantly reduced AFB_1_-induced increases in LPO and Fe^2+^ levels, suppressed oocyte apoptosis, and attenuated mitochondrial cristae loss and membrane integrity disruption. The protective effects of MYLS22 and PRGL493 were comparable to those of QT and Fer-1, indicating that OPA1 and ACSL4 are central to AFB_1_-triggered oocyte damage and ferroptosis. Previous studies indicate that OPA1 directly or indirectly modulates lipid metabolism [32, 63], while ACSL4 upregulation disrupts mitochondrial respiration and promotes mitochondria-associated ferroptosis [64, 65]. To further elucidate the role of the OPA1/ACSL4 axis in QT-mediated protection, we performed HDOCK protein–protein docking analysis, which revealed a direct interaction between OPA1 and ACSL4 stabilized by hydrogen bonds and other residue contacts, with a high docking score of –292.97. This finding is consistent with Shi et al. [33], who reported that chlorantraniliprole (CAP) disrupts OPA1 and ACSL4 expression, inducing mitophagy and ferroptosis in grass carp hepatocytes, though a direct OPA1–ACSL4 interaction was not explored. Cross-inhibition experiments further confirmed that PRGL493-mediated ACSL4 inhibition significantly reduced *OPA1* mRNA levels, while MYLS22-mediated OPA1 inhibition lowered *ACSL4* mRNA expression in AFB_1_-exposed oocytes. Additionally, MYLS22 and PRGL493 treatment enhanced the expression of antioxidant genes (*CAT* and *GPX7*) and modulated iron homeostasis regulators (*NCOA4, FTH1, SLC11A2*), reducing AFB_1_-induced Fe^2^⁺ accumulation with efficacy similar to QT and Fer-1. We also observed that AFB_1_ exposure increased *TP53* expression and decreased the *BCL2/BAX* ratio, effects reversed by co-treatment with QT, MYLS22, PRGL493, or Fer-1, indicating suppression of pro-apoptotic p53 and enhancement of anti-apoptotic Bcl-2/Bax expression. This suggests crosstalk or synergy between AFB_1_-induced ferroptosis and the apoptotic pathway, consistent with reports that ferroptosis and apoptosis can interconvert or co-activate under specific conditions [66–68]. For example, Wu et al. [66], Di et al. [67], and Zhang et al. [68] demonstrated that sodium citrate, oxaliplatin, and manganese promote both apoptosis (via Caspase3/9 activation, BAX upregulation, BCL2 downregulation) and ferroptosis (via elevated Fe^2+^, LPO, MDA, and ROS). The above results indicate, OPA1 and ACSL4 were found to interact and function cooperatively, driving mitochondrial dysfunction and membrane lipid peroxidation. ACSL4-mediated lipid metabolic disorder further activates the NCOA4-FTH1 axis to release iron ions and enhances SLC11A2-mediated iron uptake, indicating that AFB_1_ induces oocyte damage and triggers non‑canonical ferroptosis via the OPA1/ACSL4/NCOA4 pathway. Concurrently, quercetin concomitantly inhibits ferroptosis by targeting and correcting the aberrant expression of OPA1 and ACSL4, operating through mitochondrial function preservation, lipid peroxidation inhibition, and iron metabolism remodelling.

## Conclusion

The results of this study indicate that quercetin, by modulating the OPA1/ACSL4 pathway, alleviates lipid peroxidation and inhibits AFB_1_-induced ferroptosis in ovarian oocytes, thereby improving mitochondrial structure and function, maintaining iron ion homeostasis, mitigating endoplasmic reticulum stress and early apoptosis, and ultimately promoting oocyte maturation.

## Supplementary Information

Additional file 1: Fig. S1. AFB_1_ induces non-canonical ferroptosis. Table S1. Primer sequences used for quantitative PCR.

## Data Availability

The datasets used and/or analysed during the current study are available from the corresponding author on reasonable request.

## Abbreviations

ACSL4: Acyl-CoA synthetase long-chain family member 4
AFB_1_: Aflatoxin B_1_
AK2: Adenylate kinase 2
BAX: BCL2-associated X protein
BCL2: B-cell lymphoma 2
CAT: Catalase
COCs: Cumulus-oocyte complex
COX15: Cytochrome c oxidase assembly factor COX15
DECR1: 2,4-Dienoyl-CoA reductase 1
ER: Endoplasmic reticulum
ERP29: Endoplasmic reticulum protein 29
FDXR: Ferredoxin reductase
Fer-1: Ferrostatin-1
FH: Fumarate hydratase
FTH1: Ferritin heavy chain 1
GPX4: Glutathione peroxidase 4
GPX7: Glutathione peroxidase 7
GSH: Glutathione
HK2: Hexokinase-2
HSP90B1: Heat shock protein 90 beta family member 1
LPO: Lipid peroxidation
MMP: Mitochondrial membrane potential
NCOA4: Nuclear receptor coactivator 4
NNT: Nicotinamide nucleotide transhydrogenase
OPA1: Optic atrophy 1
TP53: Tumor protein 53
PB1: The first polar body
PRM: Parallel reaction monitoring
PRO: Proteomics
PUFA: Polyunsaturated fatty acid
qPCR: Quantitative PCR
QT: Quercetin
ROS: Reactive oxygen species
SLC11A2: Solute carrier family 11 member 2
SLC3A2: Solute carrier family 3 member 2
SOD3: Superoxide dismutase 3
TEM: Transmission electron microscopy
TFRC: Transferrin receptor
