# Supplementary material for: Quercetin ameliorates aflatoxin B1-induced non-canonical ferroptosis in ovine oocytes through the OPA1/ACSL4 pathway
Source: J Anim Sci Biotechnol. 2026 Jun 17;17:123. doi: 10.1186/s40104-026-01454-3 (PMC13274008; doi:10.1186/s40104-026-01454-3)
Supplement: Supplementary file 1 — Additional file 1: Fig. S1. AFB1 induces non-canonical ferroptosis. Table S1. Primer sequences used for quantitative PCR. [file 40104_2026_1454_MOESM1_ESM.docx]

**Quercetin ameliorates aflatoxin B_1_-induced non-canonical ferroptosis in ovine oocytes through the OPA1/ACSL4 pathway**

**Supplementary materials:**

**1. QT protects against AFB1-induced oocyte damage through a non-canonical ferroptosis mechanism**

Canonical ferroptosis is primarily regulated by glutathione peroxidase 4 (GPX4) and transferrin receptor 1 (TFRC/TFR1), which the decrease in GPX4 activity and the increase in TFRC expression lead to an increase in ROS and the accumulation of intracellular Fe^2+^, resulting in ferroptosis. However, in our study, the proteomic (PRO) and parallel reaction monitoring (PRM) analyses both identified that the expressions of GPX4 and TFRC proteins did not follow the canonical ferroptosis pathway (Fig. S1A–D). The results of proteomic (PRO) analysis showed that the expression of GPX4 was not significantly different in each group, while TFRC was down-regulated in the AFB_1_ group. The results of parallel reaction monitoring (PRM) for quantitative detection of GPX4 and TFRC showed that the expression of GPX4 was not significantly different in each group, and the expression of TFRC in the AFB_1_ group was abnormally down-regulated by 3.7 times compared to the control group and 4.85 times compared to the AFB_1_ + QT group, which are consistent with the proteomic results (Fig. S1A–D). Thus, we suggest that QT ameliorates AFB_1_-induced iron-dependent cell death in ovine oocytes via a non-canonical ferroptosis pathway.

As TFRC is a key iron import receptor, its suppression likely disrupted iron homeostasis, potentially triggering compensatory iron uptake via solute carrier family 11 member 2 (SLC11A2). This would lead to an expanded labile iron pool and enhanced lipid peroxidation. Immunofluorescence staining and qPCR confirmed significantly reduced FTH1 levels in AFB_1_‑treated oocytes, whereas mRNA levels of *NCOA4*, which promotes ferritin degradation, and *SLC11A2*, an iron importer, were markedly elevated (Fig. S1E–I). *FTH1* sequesters Fe^2+^ as Fe^3+^, limiting Fenton reaction substrates, while *NCOA4*‑mediated ferritinophagy and *SLC11A2* activity increase cytosolic Fe^2+^, driving ferroptosis. QT co‑treatment effectively reversed these changes, restoring *FTH1* expression, suppressing *NCOA4* and *SLC11A2*, and reducing intracellular free iron. Furthermore, *SLC3A2* mRNA levels, which are often linked to GPX4 function, showed no significant change after AFB_1_ exposure (Fig. S1J), supporting the GPX4‑independent nature of this pathway. Together, these results demonstrate that AFB_1_ triggers non‑canonical ferroptosis in ovine oocytes primarily via NCOA4‑mediated ferritin degradation and SLC11A2‑driven iron influx, rather than through GPX4 inactivation or TFRC upregulation. QT mitigates this damage by normalizing iron‑regulatory gene expression and promoting iron storage, thereby suppressing ferroptosis.


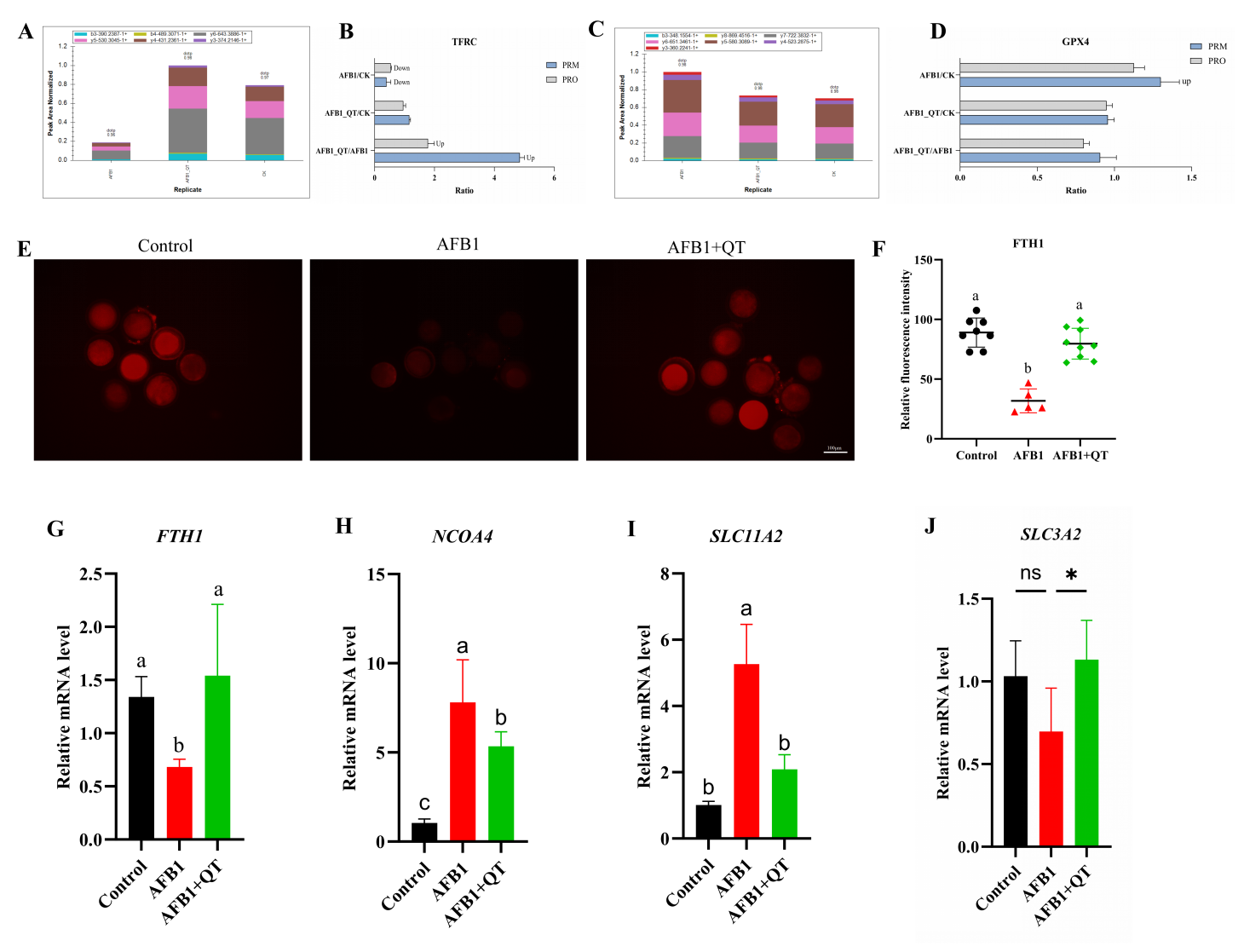


**Fig. S1** AFB_1_ induces non-canonical ferroptosis **A** Representative images of the fragment ion peak area distribution for the unique peptide YIIVGAQR of TFRC protein in the control, AFB_1_, and AFB_1_+QT groups. **B** Ratio values of TFRC protein from PRO and PRM analyses among different groups. **C** Representative images of the fragment ion peak area distribution for the unique peptide EFAAGYNVK of GPX4 protein among different groups. **D** Ratio values of GPX4 protein from PRO and PRM analyses among different groups. **E** Immunofluorescence staining of FTH1. **F** Relative fluorescence intensity values of FTH1. **G–J** *FTH1, NCOA4, SLC11A2* and *SLC3A2* mRNA levels among different groups. Building upon the initial proteomics (PRO) dataset, parallel reaction monitoring (PRM) was employed to further validate differential protein expression among different groups. Protein expression trends between the two treatment groups are expressed as mean values derived from PRO and PRM analyses, respectively. *n*=45

**2. RNA extraction and qPCR detection**

Total RNA isolated from 30–40 oocytes per group was extracted with the EASYspin Plus Ultra-micro RNA Rapid Extraction Kit (RN68, Aidlai Biology, Beijing, China). The synthesis of complementary DNA (cDNA) was executed with the PrimeScript™ FAST RT Reagent Kit, which includes a gDNA Eraser (RR092S, Takara Holdings Inc, Kyoto, Japan). For quantitative PCR (qPCR), the TB Green® Premix Ex Taq™ II (Tli RNaseH Plus) mixture (RR820A, Takara Holdings Inc, Kyoto, Japan) was used on a CFX ConnectTM Optics Module real-time fluorescence quantitative PCR system. The primer sequences can be found in Table 1 below. The data analysis involved the application of the 2^−ΔΔCt^ method.

**Table S1** Primer sequences used for quantitative PCR

| **Gene** | **Primer sequence** | **Fragment size, bp** |
| --- | --- | --- |
| *OPA1* | F-5'-GGACTTTTTCACCACAGGTTC-3' | 108 |
|  | R-5'-AGAATGAGCTCACCAAGCAGA-3' |  |
| *ACSL4* | F-5'-CACTCTCTGACCAGTCCAGC-3' | 82 |
|  | R-5'-ACAGCAGCCATGAGTGTAGG-3' |  |
| *CAT* | F-5'-AGGAGAAACCTAAGAGCAGT-3' | 90 |
|  | R-5'-CCCCCGACCCTCAGATTA-3' |  |
| *P53* | F-5'- CAGGGCTCATTCTAGCCACCT-3' | 91 |
|  | R-5'-TGAGTCAGGCCCTTCTCTCTT-3' |  |
| *Bcl2* | F-5'-CCTGTGGATGACCGAGTACCT-3' | 81 |
|  | R-5'-AGCTCCACAAAGGCATCCCAG-3' |  |
| *Bax* | F-5'-AGCTCTGAGCAGATCATGAAGA-3' | 60 |
|  | R-5'-TCGATCCTGGATGAAACCCTG-3' |  |
| FTH1 | F-5'-GTCTACCTGTCCATGGGAGC-3' | 85 |
|  | R-5'-AGCCACATCATCACGGTCAA-3' |  |
| NCOA4 | F-5'-GCCGTGACTTACGGAGCAAG-3' | 93 |
|  | R-5'-ATCACAGCACCTCGAAAGGG-3' |  |
| *SLC11A2* | F-5'-ACCGCAGAGTCCTGAATCCT-3' | 81 |
|  | R-5'-GGTATCTTCTGTTCAGGACCCAA-3' |  |
| *SLC3A2* | F-5'-TCAGCGAGGATCGGCTTTTG-3' | 82 |
|  | R-5'-TCCTTGGTGGGTTCAAGCAG-3' |  |
| *GPX7* | F-5'-CGACAGCAACAAGGAGATCG-3' | 135 |
|  | R-5'-TCCTTCCCAGAAGTCTCAGTCA-3' |  |
| *β-actin* | F-5'-CCATCGGCAATGAGCGGTTCC-3' | 146 |
|  | R-5'-CGTGTTGGCGTAGAGGTCCTTG-3' |  |
